# Rewiring reward in addiction: a systematic review of human evidence for deep brain stimulation and lesion approaches in substance use disorders

**DOI:** 10.1007/s00701-026-06979-0

**Published:** 2026-07-14

**Authors:** Yaxel Levin-Carrion, Juan Miguel Riestra, Taylor Campbell, Kevin Titkov, Naeem Gibson, Diana B. Riestra, Sedra T. Alabed, Shreya Meda, Ashley Raghu, Steve Gad, Daniel Brenner, Alejandro Pando, Phillip Bempong, Jonathan H. Sherman, Petros Levounis, Robert E. Gross

**Affiliations:** 1 Rutgers New Jersey Medical School, Department of Neurological Surgery, Newark, NJ USA; 2 Rutgers New Jersey Medical School, Department of Psychiatry, Newark, NJ USA; 3 Department of Neurosurgical Oncology, Rutgers Cancer Institute of New Jersey, New Brunswick, NJ USA; 4 Department of Neurosurgery, Rutgers University, 185 S Orange Ave, Newark, NJ 07103 USA

**Keywords:** Deep brain stimulation (DBS), Substance use disorder (SUD), Nucleus accumbens (NAc), Alcohol use disorder, Reward circuitry, Ablation

## Abstract

**Objective:**

Neurosurgical modulation of reward circuitry has been explored for severe, treatment-refractory substance use disorders (SUDs), yet the human literature remains fragmented across targets, modalities, and outcomes, limiting target selection and future trial design. We systematically reviewed human clinical evidence for deep brain stimulation (DBS) and lesion approaches targeting reward network nodes for SUDs, and summarized convergent preclinical findings to identify where the strongest clinical signal exists and to provide a framework for mechanism-informed future trials.

**Methods:**

We systematically searched PubMed, PubMed Central, Embase, and Scopus (inception-September 9, 2025) for human DBS/ablation studies in primary SUDs targeting addiction-relevant nodes (e.g., nucleus accumbens [NAc], ventral capsule/ventral striatum/anterior limb of internal capsule [ALIC], subthalamic nucleus) and reporting extractable outcomes; in vivo addiction-model animal studies were included for mechanistic context. Findings were synthesized narratively.

**Results:**

Forty-seven studies met inclusion criteria. The clearest controlled human signal came from bilateral NAc DBS in severe alcohol use disorder, with additional promising but less mature findings in heroin/opioid DBS. In the only randomized delayed-activation trial of bilateral NAc DBS for severe alcohol use disorder (*n* = 12), abstinent days increased from 27.6% to 56.0% at 6 months (*p* = 0.014) and 74.2% at 18 months (*p* = 0.004), and heavy drinking days decreased from 69.6% to 47.4% (*p* = 0.020) and 35.5% (*p* = 0.031). Craving improved (OCDS 21.0 to 10.5 at 6 months, *p* = 0.014; 5.9 at 18 months, *p* = 0.008), with blinded-phase separation favoring earlier stimulation for abstinent/heavy drinking days (*p* = 0.048). An open-label alcohol DBS pilot reported drinks/day decreasing from 10.4 to 2.7 at 12 months (*p* < 0.05) and OCDS from 28.7 to 8.3 (*p* < 0.05). In heroin use disorder DBS targeting NAc ± ALIC (*n* = 8), 5/8 remained abstinent for > 3 years; craving VAS decreased from 7.6 to 1.4 at 3 months and 0.8 at 24 months (*p* < 0.001), with depressive symptoms improving (HDRS 12.4 to 3.4; *p* = 0.001). In a polysubstance feasibility study (*n* = 4), two participants maintained abstinence for > 1150 and > 520 days; craving decreased in individuals (opioid craving *p* = 0.036; benzodiazepine *p* = 0.001). Biomarker-guided opioid DBS reduced cue-induced craving (6.93 vs 3.09; *p* < 0.001). Ablation cohorts showed short-term improvements (alcohol NAc ablation: 3/12 relapsed; dependence severity 39.08 to 9.25, *p* < 0.01), but longer-term relapse and neuropsychological tradeoffs were reported (4-year abstinence 53.8% overall). Preclinical studies highlighted strong target-and state-dependence, including paradoxical relapse augmentation in alcohol-dependent models.

**Conclusion:**

DBS and lesion approaches should be interpreted as distinct neurosurgical modalities rather than a single therapeutic class. The strongest controlled human signal currently comes from bilateral NAc DBS in severe alcohol use disorder, with additional but lower-certainty DBS evidence in opioid/heroin and polysubstance use disorders. Lesion approaches provide historical and mechanistic support for the relevance of ventral striatal circuitry, but their irreversibility, heterogeneous follow-up, and neuropsychological tradeoffs require a higher evidentiary and ethical threshold. Overall, current data support clinical plausibility and trial-design rationale, but not definitive efficacy across SUD subtypes

**Supplementary Information:**

The online version contains supplementary material available at https://doi.org/10.1007/s00701-026-06979-0.

## Introduction

Substance use disorders (SUDs) are chronic, relapsing neuropsychiatric conditions characterized by maladaptive reinforcement learning, impaired inhibitory control, and persistent cue reactivity [28, 51]. Despite effective behavioral and pharmacologic therapies for many patients, a subgroup of patients with SUD develop severe, refractory illness with repeated relapse, medical complications, and elevated mortality [39, 49]. For these individuals, the urgency of innovation has led to renewed interest in neurosurgical interventions, particularly deep brain stimulation (DBS). DBS can modulate circuit-level drivers of craving, motivational attentional bias from drug-related stimuli, and compulsive drug seeking [3, 12, 65]. Addiction networks are well established, principally the interconnected mesocorticolimbic networks anchored in the nucleus accumbens (NAc), ventral capsule/ventral striatum (VC/VS), medial prefrontal cortex (mPFC), anterior cingulate cortex (ACC), amygdala, hippocampus, and subthalamic nucleus (STN) [28, 29]. These regions participate in reward valuation, habit formation, affect regulation, and cognitive control [28, 29, 57]. DBS has transformed care for common movement disorders and is increasingly being pursued as a therapy for refractory psychiatric illness; the relative reversibility of DBS makes it an appealing alternative to destructive procedures for some patients [17, 27].

Addiction adds treatment barriers: variable motivation, relapse risk, and family or system pressures. These factors elevate the ethical burden of consent and long-term monitoring. DBS was first explored as a potential addiction therapy in 2007 by Kuhn et al., after bilateral NAc stimulation was associated with marked remission of comorbid alcohol dependence [30].

However, DBS had been applied to addiction-relevant circuits much earlier, including the STN and VC/VS, with observations that compulsive and addictive behaviors could be either ameliorated or exacerbated depending on target, stimulation, and medication context [48, 50, 62]. Ablative procedures, particularly NAc lesioning, have been used historically in China and are still practiced to a limited extent for severe, treatment-refractory opioid dependence, though concerns persist regarding neuropsychiatric side effects and the ethics of irreversible interventions [11, 32]. In parallel to these clinical studies, controlled animal studies have provided mechanistic insights into DBS models including identifying parameter and disease-state dependencies, such as paradoxical worsening of relapse behaviors depending on timing, stimulation frequency, and dependence phenotype.

The aim of this systematic review is threefold. First, we synthesize clinical evidence for neurosurgical approaches to SUDs, while explicitly distinguishing programmable DBS from irreversible lesion/ablation ablative procedures across alcohol, opioids, stimulants, nicotine, and polysubstance use. Second, we integrate preclinical findings to clarify plausible mechanisms and inform target selection. Third, we analyze ethical and trial-design issues unique to neurosurgical interventions in addiction and propose a roadmap for future sham-controlled, circuit-guided trials.

## Methods

### Protocol and reporting

**Fig. 1:**
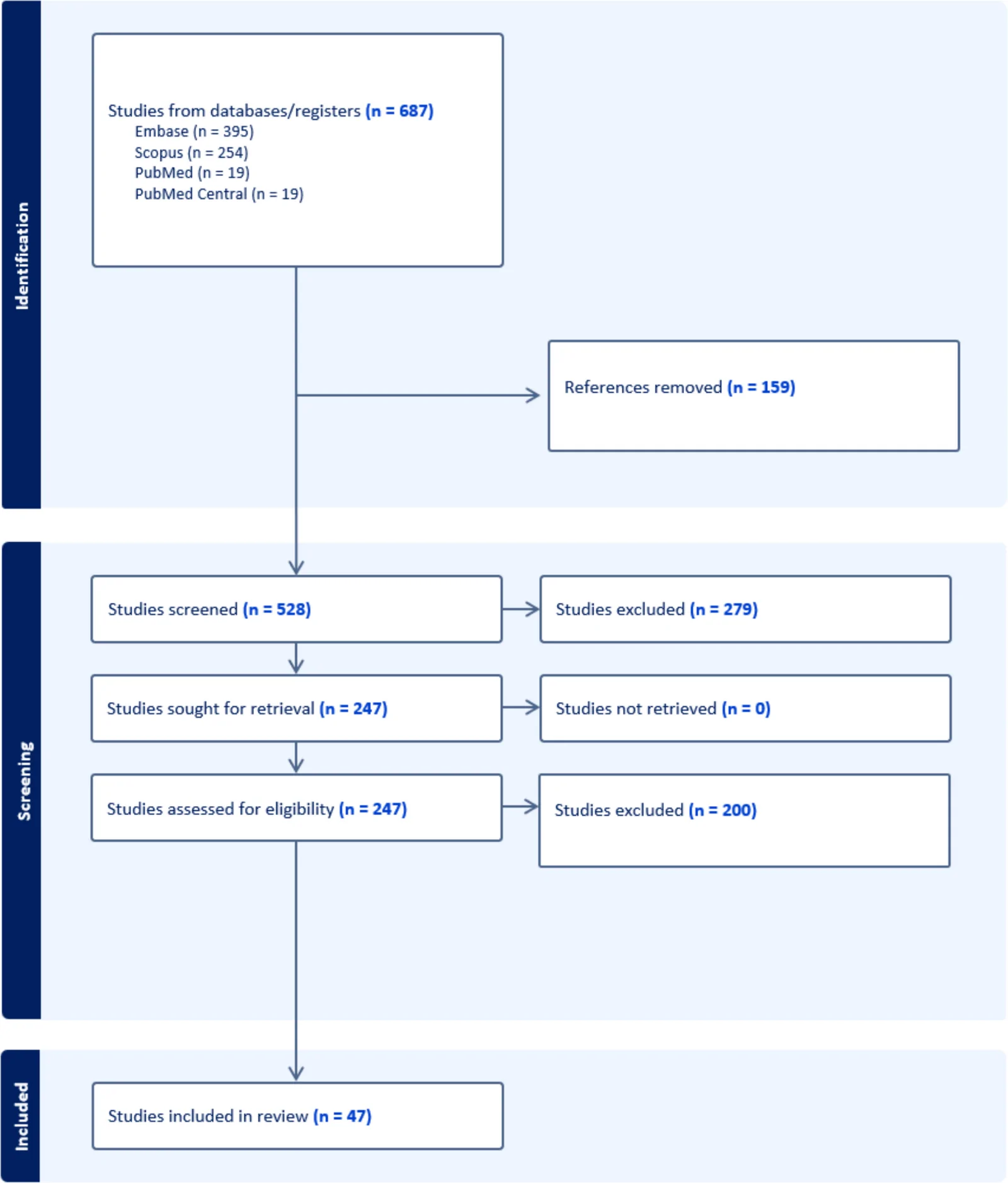
PRISMA flowchart

This review was conducted in accordance with PRISMA 2020 guidance. An a priori protocol specified objectives, eligibility criteria, outcomes, and analytic approach, and a PRISMA flowchart was created for the screened studies (Fig. 1).

### Information sources and search strategy

We searched four databases: PubMed, PubMed Central (PMC), Embase, and Scopus. Searches were conducted from database inception through September 9th, 2025. No restrictions were applied at the search stage based on substance class; all SUDs were eligible if they involved an addictive substance and met the intervention/target criteria. Full search terms are available in Supplemental Table 1.

### Eligibility criteria

Human studies were eligible if they met all of the following: (1) Population: participants with a primary diagnosis of an SUD (including alcohol, opioids/heroin, stimulants such as cocaine or methamphetamine, nicotine/tobacco, or polysubstance use); (2) Intervention: invasive neurosurgical intervention intended to treat SUD symptoms, including DBS, stereotactic lesioning/ablation, or combined procedures; (3) Target: at least one addiction-relevant target in the reward/control circuitry, including NAc, VC/VS/anterior limb of the internal capsule (ALIC), STN or medial forebrain bundle (or clearly adjacent fiber pathways intended to modulate the same network); and (4) Outcomes: reported at least one addiction-relevant efficacy or safety endpoint with extractable data, including binary/nominal outcomes (e.g., abstinent vs relapsed; adverse events), time-to-event outcomes (e.g., time to relapse), count/continuous outcomes (e.g., days using, drinks/day, quantity/frequency), or ordinal/continuous scale scores (validated or investigator-defined). Narrative outcomes were eligible only if they specified a clear status or direction of change at a defined follow-up. We included randomized trials, prospective cohorts, retrospective series, and case reports. We also included conference abstracts only if they reported original human neurosurgical SUD data with enough methodological detail to extract target, sample, and outcomes; otherwise they were excluded. We excluded studies if the neurosurgical intervention was performed for a different primary indication (e.g., movement disorder or psychiatric disorder) and SUD outcomes were not reported or not attributable; the intervention was non-invasive neuromodulation only (TMS, tDCS, etc.); or the study lacked sufficient data to confirm the target/intervention or lacked outcomes relevant to SUD. Preclinical studies were eligible if they met all of the following: (1) Model: in vivo animal studies (rodent or other) using an addiction-relevant paradigm (self-administration, conditioned place preference (CPP), extinction/reinstatement, relapse-like drinking, progressive ratio/motivation measures); (2) Intervention: DBS or invasive stimulation/lesion manipulation applied to addiction-relevant targets; (3) Outcomes: reported at least one quantitative addiction-relevant behavioral endpoint (e.g., intake, lever presses, reinstatement responding, preferences scores, breakpoints). Preclinical studies were included to support mechanistic interpretation and to summarize parameter- and state-dependent effects relevant to human trial planning. Human clinical evidence was treated as the primary evidence base for efficacy and safety. Preclinical studies were prespecified as a mechanistic-context layer and were synthesized separately; they were not used to infer clinical efficacy. Accordingly, animal findings were used to identify candidate mechanisms, target subregions, stimulation parameters, and state-dependent effects that may inform human trial design.

### Study selection

All references were imported into Covidence for management of deduplication and screening. Covidence’s automated deduplication was used first, followed by manual spot checks for remaining duplicates (e.g., conference abstract vs full publications). Titles/Abstracts were screened by two independent reviewers using prespecified inclusion/exclusion criteria. Full-text review was then completed by two independent reviewers for studies advanced after title/abstract screening. Conflicts were resolved by consensus review. When multiple publications appeared to report overlapping participants, cohorts, or extended follow-up from the same center, we treated the most complete peer-reviewed report or the longest follow-up report as the primary source for cohort-level interpretation. Earlier reports, conference abstracts, or secondary analyses were retained only when they contributed non-overlapping information, such as programming details, mechanistic biomarkers, adverse events, imaging findings, or longer-term follow-up. Conference abstracts were excluded from primary interpretation when a peer-reviewed full report of the same cohort was available.

### Data extraction

Extraction was performed using a standardized template and checked for consistency across reviewers. For clinical studies, we extracted study characteristics (country, design, single vs. multicenter, sample size, setting, follow-up duration, funding and conflicts), clinical characteristics (primary substance category, secondary substances, severity metrics, years of use, baseline use metrics, prior treatment failures, comorbid psychiatric/neurologic conditions, age and sex), and intervention details (surgery type, primary target, laterality, targeting approach, device manufacturer, stereotactic coordinates when reported, and post-operative imaging verification). For DBS studies, stimulation and programming variables were abstracted when available (time to programming start, number of programming sessions, stimulation mode, frequency, pulse width, amplitude, duty cycle, and whether parameters were changed over time). We also extracted trial design features (control type, randomization and allocation concealment when applicable), concurrent treatments (psychosocial care and medications), and ethics/safety reporting fields (consent safeguards and adverse event monitoring). For preclinical studies, we extracted model characteristics (species/strain/sex, housing, randomization/blinding, sample size), addiction paradigm and substance exposure protocol, surgical/stimulation details (target, stereotactic coordinates, verification), stimulation parameters and schedule, and whether pharmacologic manipulations were used.

### Data synthesis

Given substantial heterogeneity in population characteristics, neurosurgical targets, stimulation parameters, follow-up duration, and outcome definitions, we performed a narrative synthesis rather than a meta-analysis. We grouped clinical results by primary substance class (alcohol, opioids/heroin, stimulants, polysubstance) and by target (NAc/VC/VS pathways vs. alternative targets such as STN or cortical nodes), then summarized patterns in efficacy signals and adverse events. Preclinical studies were synthesized separately and were used to highlight mechanistic plausibility and parameter/state dependence (e.g., stimulation timing relative to extinction/reinstatement, dependent vs. nondependent phenotypes). DBS and lesion/ablation studies were not pooled and were interpreted as distinct modalities. DBS was considered programmable and reversible at the level of stimulation delivery, whereas lesion approaches were considered irreversible interventions with distinct safety, ethical, and neuropsychological implications. We therefore summarized DBS and ablation evidence separately, then compared them only at the level of circuit rationale, endpoints, and trial-design implications.

## Results

### Study characteristics

47 studies (mixed clinical and preclinical) were included. Targets included NAc core/shell, VC/VS or ALIC, ACC/mPFC subregions, and STN. Clinical evidence (Table 1) consisted of one randomized study, 9 small prospective trials/pilots, and case reports (10)/series (5). Ablation studies were predominantly from NAc lesioning cohorts, with surgical targeting and hardware details summarized in Table 2. Clinical DBS programming and stimulation parameters are summarized in Table 3. Relevant clinical and preclinical study characteristics, design features, concomitant care, safety monitoring, and stimulation protocols are summarized in Tables 4, 5, 6. To make the evidence hierarchy explicit, randomized or blinded human DBS data were interpreted as the highest level of evidence, followed by prospective open-label DBS cohorts, retrospective series, single-case reports, uncontrolled lesion cohorts, and preclinical studies. Preclinical studies were considered mechanistic and translational rather than evidence of clinical efficacy. Under this framework, bilateral NAc DBS for severe alcohol use disorder represents the strongest clinical signal, while opioid/heroin DBS remains promising but lower certainty, stimulant DBS remains very sparse, and ablation studies are best viewed as historical and mechanistic comparators rather than equivalent alternatives to DBS.

**Table 1:** Included clinical studies and participant context

Study	Country	Design	*n*	Primary SUD	Setting	Follow-up (max)
Gao, 2003	China	Prospective case series	28	OUD (opium, heroin, and other opioids)	Mixed (inpatient detoxification preoperatively; outpatient follow-up postoperatively)	Mean 15 months (range 2.5–26 months)
Muller, 2009	Germany	Case series	3	AUD	Mixed (inpatient detoxification preoperatively; outpatient follow-up postoperatively)	18 months (Patient 1); 12–15 months (others)
Wu, 2010	China	Prospective case series	12	AUD	Mixed (inpatient detoxification preoperatively; community-based follow-up postoperatively)	Mean 16.58 months (maximum 27 months)
Kuhn, 2011	Germany	Single-case report	1	AUD	Mixed (inpatient surgery; monitored outpatient follow-up postoperatively)	12 months
Zhou, 2011	China	Single-case report	1	OUD (heroin)	Mixed (ICU detoxification preoperatively; inpatient and outpatient follow-up postoperatively)	72 months
Fang, 2012	China	Observational intraoperative stimulation study	61 stimulated (70 recruited)	OUD (heroin)	Inpatient neurosurgical setting	NA (intraoperative only)
Heldmann, 2012	Germany	Single-case experimental study	1	AUD (severe, treatment-refractory)	Inpatient surgery; follow-up setting not reported	18 months
Valencia-Alfonso, 2012	Netherlands	Single-case report	1	OUD (heroin)	Mixed (inpatient surgery; outpatient follow-up postoperatively)	10 months
Li, 2013	China	Clinical trial with retrospective follow-up and randomized lesion-method comparison	272 operated; 93 long-term follow-up; 78 lesion-method RCT	OUD (heroin)	Mixed (hospital-based follow-up, home visits, and rehabilitation-center follow-up postoperatively)	60 months
Voges, 2013	Germany	Open-label case series	5	AUD	Mixed (inpatient detoxification preoperatively; outpatient follow-up postoperatively)	47 months (range 31–47 months; mean 38)
Wang, 2013	China	Observational follow-up study	18 included (42 original surgeries)	OUD (heroin)	Mixed (inpatient surgery; outpatient and telephone follow-up postoperatively)	103 months
Muller, 2016	Germany	Pilot case series	5	AUD	Mixed (inpatient detoxification preoperatively; long-term outpatient follow-up postoperatively)	96 months
Goncalves-Ferreira, 2016	Portugal	Single-patient pilot study with double-blind crossover	1	Stimulant (cocaine)	Mixed (inpatient surgical admission; outpatient follow-up postoperatively)	30 months
Chen, 2019	China	Open-label pilot study	8	OUD (heroin)	Mixed (inpatient surgery and detoxification; outpatient follow-up postoperatively)	40.6 ± 3.36 months
Ge, 2019	China	Case report	2	Stimulant (methamphetamine)	Outpatient follow-up	18 months (Patient A); 30 months (Patient B)
Zhang, 2019	China	Case report	1	Stimulant (methamphetamine)	Mixed (inpatient surgery; outpatient follow-up postoperatively)	12 months
Zhang, 2020	China	Case report	1	OUD (heroin)	Mixed (inpatient surgery; outpatient counseling and testing postoperatively)	24 months
Zhu, 2020	China	Case report	1	Polysubstance (bucinnazine, morphine, hypnotics (zopiclone, alprazolam))	NR	12 months
Mahoney, 2021	United States	Single-case report	1	OUD (severe, treatment-refractory)	Mixed (inpatient phases I–IV; outpatient follow-up in phase V)	12 months
Davidson, 2022	Canada	Prospective open-label pilot trial	6	AUD (severe, refractory)	Mixed (inpatient surgery; outpatient follow-up postoperatively)	12 months
Bach, 2023	Germany	Double-blind randomized controlled trial	12	AUD	Mixed (inpatient surgery; outpatient follow-up postoperatively)	18 months
Rezai, 2023	United States	Prospective open-label single-arm trial	4	OUD (severe, treatment-refractory)	Mixed (4–6-week inpatient baseline, implantation, and titration; outpatient follow-up thereafter)	38 months (Patient 1); 17 months (Patient 3)
Vorspan, 2023	France	Single-case report with double-blind crossover	1	Stimulant (cocaine)	Mixed (inpatient treatment; outpatient follow-up and social housing support)	24 months
Qiu, 2024	United States	Single-patient case study	1	OUD	Mixed (inpatient externalization and assessment; outpatient follow-up postoperatively)	6 months
Lipsman 2025	Canada	Phase I pilot trial	6	AUD (severe, refractory)	NR	12 months

*NR* not reported, *NA* not applicable. All values are extracted as reported in the source studies

*AUD* alcohol use disorder, *OUD* opioid use disorder

**Table 2:** Surgical targeting and hardware details in clinical studies

Study	Surgery type	Target	Laterality	Targeting method	Coordinates (x,y,z) [mm]	Verification imaging	Ablation method	Lesion volume (mm^3^)
Gao, 2003	Ablation (stereotactic radiofrequency lesioning)	NAc	Bilateral	Anatomical targeting with CT-based stereotaxy (CRW frame)	x = 4–10 mm lateral to midline; y = 19–23 mm anterior to AC-PC midpoint; z = 7–9 mm inferior to AC-PC line	Preoperative CT; postoperative verification NR	Radiofrequency lesioning at 85 °C	NR
Muller, 2009	DBS	NAc (shell distally; core proximally)	Bilateral	Anatomical targeting with MRI/CT fusion and atlas-based coordinates	x = 6.5 mm lateral to midline; y = 2.7 mm rostral to AC; z = 4.5 mm ventral to AC	Intraoperative stereotactic X-ray; CT/MRI fusion	NA (DBS)	NA
Wu, 2010	Ablation (radiofrequency lesioning)	NAc (core region)	Bilateral	Anatomical (AC-PC coordinates + MRI guidance + macrostimulation)	x = 4–10 mm lateral to midline; y = 19–23 mm anterior to AC-PC midpoint; z = 7–9 mm inferior to AC-PC line	MRI for targeting; postoperative verification NR	Radiofrequency (85 °C, 1.8 mm electrode)	Maximum diameter 0.5 cm; exact volume NR
Kuhn, 2011	DBS	NAc	Bilateral	Stereotactic neurosurgery	x = NR; y = NR; z = NR	NR	NA (DBS)	NA
Zhou, 2011	DBS	NAc	Bilateral	Stereotactic anatomical targeting (coordinates provided)	x = 6.5 mm lateral to midline; y = 7.5 mm anterior to AC; z = 6 mm inferior to AC-PC line	Postoperative MRI and X-ray	NA (DBS)	NA
Fang, 2012	Stereotactic radiofrequency ablation (post-stimulation)	NAc and amygdala (AMY)	Bilateral	Anatomical (CT/MRI + Schaltenbrand-Wahren atlas)	NAc: x = ± 10 mm; y = + 1 mm; z = − 2 mm AMY: x = ± 21 mm; y = + 8 mm; z = − 12.5 mm	NR	Radiofrequency heat lesioning	NR
Heldmann, 2012	DBS	NAc (current spread to BNST and VP)	Bilateral	Stereotactic anatomical targeting with intraoperative X-ray and postoperative CT fused to MRI	x = NR; y = NR; z = NR	Intraoperative stereotactic X-ray; postoperative CT/MRI fusion	NA (DBS)	NA
Valencia-Alfonso, 2012	DBS	NAc + adjacent anterior limb of internal capsule (ALIC)	Bilateral	Anatomical (stereotactic coordinates provided)	x = 7 mm lateral; y = 3 mm anterior to AC border; z = 4 mm inferior	NR	NA (DBS)	NA
Li, 2013	Bilateral stereotactic radiofrequency ablation	NAc (posterior NAc; shell + core depending on group)	Bilateral	Anatomical MRI + Schaltenbrand atlas	x = 5.5–6.5 mm lateral to AC; y = 5.0–6.0 mm anterior to AC; z = 5.5–6.5 mm inferior to AC	Postoperative MRI	Radiofrequency (80 °C × 60 s)	NR
Voges, 2013	DBS	NAc, caudo-medial region (shell remnant)	Bilateral	Anatomical MRI-based targeting + stereotactic CT fusion	x = 6–8 mm lateral to midline; y = 2 mm rostral to AC; z = 3–4 mm ventral to AC	Intraoperative stereotactic X-ray; postoperative CT/MRI fusion	NA (DBS)	NA
Wang, 2013	Ablation (stereotactic radiofrequency lesioning)	NAc	Bilateral	Anatomical targeting using CT coordinates + MRI confirmation	x = NR; y = NR; z = NR	Postoperative MRI	Radiofrequency (RF) lesioning	NR
Muller, 2016	DBS	NAc	Bilateral	Stereotactic neurosurgery	x = NR; y = NR; z = NR	Postoperative CT/MRI	NA (DBS)	NA
Goncalves-Ferreira, 2016	DBS	Posteromedial NAc (shell) + BNST	Bilateral	Stereotactic anatomical targeting with MRI/CT fusion; neuronavigation	x = NR; y = NR; z = NR	MRI/CT fusion; intraoperative testing	NA (DBS)	NA
Chen, 2019	DBS	NAc + ALIC (single lead)	Bilateral	Anatomical MRI-based stereotactic planning (Leksell frame + Surgiplan)	x = 6–9 mm lateral (post-op: 8–10.5 mm); y = 16–18 mm anterior (post-op: 15.5–18.5 mm); z = 4–6 mm inferior to MCP (post-op: 4.5–8.5 mm)	Postoperative 1.5 T MRI	NA (DBS)	NA
Ge, 2019	DBS	NAc + anterior limb of internal capsule (ALIC)	Bilateral	Anatomical targeting with MRI/CT coregistration	x = NR; y = NR; z = NR	MRI/CT fusion	NA	NA
Zhang, 2019	DBS	NAc + VC	Bilateral	Standard stereotactic targeting	x = NR; y = NR; z = NR	Postoperative CT; preoperative and postoperative PET	NA (DBS)	NA
Zhang, 2020	DBS	NAc	Bilateral	NR	x = NR; y = NR; z = NR	Preoperative, postoperative, and post-explant structural imaging	NA (DBS)	NA
Zhu, 2020	Combined: bilateral anterior capsulotomy + bilateral NAc DBS	NAc (DBS) + ALIC (ablative capsulotomy)	Bilateral	Anatomical targeting using MRI + CT merged in Leksell SurgiPlan	x = NR; y = NR; z = NR	Postoperative MRI and CT	Radiofrequency thermocoagulative ablation (75 °C for 60 s; 15 mm lesion length bilaterally)	NR
Mahoney, 2021	DBS	NAc/VC	Bilateral	NR	x = NR; y = NR; z = NR	Postoperative FDG-PET; CT/MRI implied	NA (DBS)	NA
Davidson, 2022	DBS	NAc/ventral striatum (VS)	Bilateral	Anatomical targeting with MRI/CT co-registration	x = NR; y≈2.5 mm rostral to AC; z = 4 mm ventral to AC	Postoperative MRI	NA (DBS)	NA
Bach, 2023	DBS	NAc (caudomedial region); contacts at ALIC border zone	Bilateral	Combined indirect stereotactic coordinates + direct MRI anatomical targeting	x = 6–8 mm lateral to midline; y = 2 mm rostral to AC; z = 3–4 mm ventral to AC-PC plane	Postoperative CT or MRI	NA (DBS)	NA
Rezai, 2023	DBS	NAc/VC	Bilateral	Anatomical targeting using 3 T MRI + StealthStation planning + microelectrode recording	x = 6–9 mm lateral to AC; y = 1–3 mm anterior to AC; z = 4–6 mm inferior to mid-commissural plane	Postoperative CT merged with preoperative MRI	NA (DBS)	NA
Vorspan, 2023	DBS	STN	Bilateral	Anatomical stereotactic targeting	x = NR; y = NR; z = NR	Postoperative MRI	NA (DBS)	NA
Qiu, 2024	DBS	NAc shell	Unilateral (left)	Anatomical + tractography-based structural connectivity analysis	x = NR; y = NR; z = NR	Postoperative CT co-registered to MRI	NA (DBS)	NA
Lipsman 2025	DBS	NAc	NR	NR	x = NR; y = NR; z = NR	Postoperative FDG-PET and fMRI	NA (DBS)	NA

*NR*  not reported, *NA* not applicable. All values are extracted as reported in the source studies

*DBS* deep brain stimulation, *NAc* nucleus accumbens

**Table 3:** Programming and stimulation parameters in clinical studies

Study	Programming start (days post-op)	Programming sessions (*n*)	Stimulation mode	Frequency (Hz)	Pulse width (μs)	Amplitude (V or mA)	Duty cycle	Parameter changes over time
Gao, 2003	NA (no DBS)	NA	NA	NA	NA	NA	NA	NA
Muller, 2009	NR	NR	Continuous	130 Hz	90 µs	Patient 1: 4.5 V; Patient 2: 3.5 V; Patient 3: 3.5 V	Continuous (no cycling reported)	Yes; Patient 2 required adjustment due to hypomania; others remained stable
Wu, 2010	NA (no DBS)	NA	NA	NA	NA	NA	NA	NA
Kuhn, 2011	NR	NR	Continuous	NR	NR	NR	NR	NR
Zhou, 2011	7 days postoperatively	NR	Continuous	145 Hz	NR	0.8 V → 2.5 V over weeks 2–5	Continuous; no cycling reported	Yes; amplitude increased from 0.8 to 2.5 V over weeks 2–5
Fang, 2012	NR	NA	Intraoperative test stimulation only (50 Hz, 2–4 V)	50 Hz	NR	2–4 V	NR	NA (single-session intraoperative stimulation)
Heldmann, 2012	7 days postoperatively	NR (initial adjustment after hypomania; long-term settings stable)	Continuous	130 Hz	90 µs	3.5 V	Continuous	Yes; early adjustment for transient hypomania, then stable
Valencia-Alfonso, 2012	7 days postoperatively	NR	Continuous	180 Hz	90 µs	3.5 V	Continuous	Yes; multiple contact configurations tested with slight voltage adjustment
Li, 2013	NA (ablation, not DBS)	NA	NA	NA	NA	NA	NA	NA
Voges, 2013	NR	NR	Continuous	130 Hz	90 µs	Initial 4.5 V (bipolar); adjusted to 3.5 V (monopolar) in 1 patient	Continuous	Yes; 1 patient switched to monopolar due to hypomania
Wang, 2013	NA (no DBS)	NA	NA	NA	NA	NA	NA	NA
Muller, 2016	NR	NR	Continuous (standard DBS)	NR	NR	NR	NR	No changes made during relapses; parameters remained constant
Goncalves-Ferreira, 2016	Day 5	NR	Continuous	Initial 130 Hz; final 150 Hz	150 µs	Initial 2 V; final: Right 3–4 V, Left 2.5–3 V	Continuous (100%)	Yes progressive adjustments; anterior limb of internal capsule contacts found ineffective
Chen, 2019	14 days	39 total across all patients during optimization; 9 additional adjustments in 3 patients	Continuous	NAc: 145 Hz; ALIC: 185 Hz	150–240 µs	NAc: 2.2–2.8 V; ALIC: 1.5–2.4 V	Continuous (no cycling reported)	Yes 39 adjustments during initial optimization; 9 later adjustments in 3 patients
Ge, 2019	14 days	NR	Continuous	150–165 Hz	210–240 µs	2.5–4.5 V	Continuous	Yes; adjustments for insomnia, hypomania, bruxism
Zhang, 2019	Initiated after postoperative CT; exact day NR; parameters adjusted during first month	NR	Continuous	130 Hz	Initially 210 µs; adjusted to 90 µs during first month	Initially 3.0 V; adjusted to 2.5 V	NR	Yes; amplitude and pulse width reduced to avoid agitation/hypomania
Zhang, 2020	NR	NR	Continuous bilateral stimulation for 1 year	NR	NR	NR	Continuous	NR
Zhu, 2020	14 days (2 weeks)	NR (parameters adjusted over 6 months)	Continuous	Initial: 160 Hz; Final: Left 145 Hz, Right 160 Hz	90 µs	Initial: 2.8 V; Final: Left 2.75 V, Right 2.70 V	Continuous (no cycling reported)	Yes adjustments over 6 months to final settings
Mahoney, 2021	NR	NR (multiple titrations; final adjustment at Week 7)	Continuous	145 Hz	90 µs	6 V	Continuous (no cycling reported)	Yes titrated through Phase IV; final parameters set Week 7
Davidson, 2022	~ 14 days postoperatively	NR (multiple visits; additional visits if deterioration)	Continuous DBS	130 Hz	90 µs	Voltage mode; titrated; example: 3.5–4.5 V; exact per patient NR	Continuous (100%)	Yes titrated based on clinical effect; alternate contacts trialed
Bach, 2023	Immediately after baseline (EARLY group) or at 6 months (LATE group)	Adjustments allowed at weeks 2 and 4; otherwise fixed protocol	Continuous monopolar stimulation (cathodic)	130 Hz	90 µs	3.5 V initial; adjustable to max 4.5 V	Continuous (no cycling reported)	Yes amplitude adjustments at weeks 2 and 4 if needed
Rezai, 2023	During inpatient titration phase; exact day NR	NR; titration occurred over 4–6 weeks inpatient	Continuous (standard DBS) NR but implied by parameters	125–145 Hz depending on participant	90–300 µs depending on participant	3.0–4.5 mA depending on participant	NR	Yes optimized during inpatient titration; some participants had adjustments during outpatient follow-up
Vorspan, 2023	First double-blind phase began 1 month post-surgery	NR (multiple adjustments described; exact count NR)	Continuous	130 Hz	60 µs	2.0 V initially; hypomania occurred at 3.0 V; later reduced to 1.25 V	Continuous (no cycling reported)	Yes voltage adjustments; progressive titration in third cross-over
Qiu, 2024	After externalization; exact days NR	NR; acute stimulation mapping performed; chronic programming established	Continuous DBS (chronic)	130 Hz	90 µs	6 mA (personalized setting)	Continuous (no cycling reported)	Yes reduced from 14.5 mA (prior) to 6 mA after biomarker-guided optimization
Lipsman 2025	NR	NR	NR	NR	NR	NR	NR	NR

*NR* not reported, *NA* not applicable. All values are extracted as reported in the source studies

*DBS* deep brain stimulation

**Table 4:** Study design, concomitant care, and safety monitoring in clinical studies

Study	Comparator	Randomization	Allocation concealment	Psychosocial care	Medications	Adverse event monitoring	Funding	Conflicts of interest
Gao, 2003	None (uncontrolled case series)	No	NA	None reported; patients returned to usual environment without structured therapy	Detoxification meds pre-op: dolantin, tramadol, methadone, scopolamine, anisodamine, chlorpromazine, phenergan	Monitored for neurological deficits, personality change, memory impairment; 4 cases temporary hypomnesia; 2 mild personality change	NR	NR
Muller, 2009	None (open-label case series)	No	NA	No additional psychotherapy post-DBS (only routine assessments)	No anti-craving meds post-DBS; acamprosate previously failed	Routine clinical follow-up; hypomania in patient 2 resolved with parameter adjustment	NR	Some authors reported Medtronic-related fees/patent involvement
Wu, 2010	None (uncontrolled case series)	No	NA	Pre-op detoxification; no structured psychotherapy reported post-op	Detox medications pre-op (NR); no maintenance meds reported	Monitored for hematoma, infection, neurological deficits; 1 case of transient hyposmia	NR	NR
Kuhn, 2011	On/off DBS comparisons at T2, T3, T4 (within-subject baseline control)	No	NA	NR	NR	Patient kept under close observation during follow-ups to prevent relapse; no surgical complications reported	Some authors received travel support, lecture fees, or research support from Medtronic AG and Advanced Neuromodulation Systems Inc	Several authors reported financial relationships with Medtronic; others reported none
Zhou, 2011	None (case report)	No	NA	None reported during follow-up (no ancillary treatment)	Detox medications during anesthesia-assisted rapid detox; none ongoing for relapse prevention	ICU monitoring, urine tests, naloxone challenge tests, psychometric scales	National Natural Science Foundation of China (Grant 30,600,634)	Authors report none
Fang, 2012	None (within-subject comparisons of stimulated vs. unstimulated states)	No	NA	NR	Detoxification medications prior to surgery; specifics NR	Vital signs monitored (heart rate, arterial pressure) during stimulation	Wu Jie-ping Medical Foundation (No. LC03002)	NR
Heldmann, 2012	Within-subject on/off comparison; no sham; PET sessions counterbalanced across days	No	NA	NR	Pre-surgery: acamprosate (failed). No ongoing MOUD or AUD meds reported post-DBS	Hypomania monitored and resolved with parameter adjustment; no other AEs reported	DFG (SFB779) to TFM, BB, HJH	Authors report none
Valencia-Alfonso, 2012	None (case report)	No	NA	NR (no concurrent therapy described during DBS phase)	Buprenorphine 6 mg during treatment episode	Weekly assessments; no specific adverse events reported	ZonMw Geestkracht Programme, Grant 60–60600-97–168	Some authors received travel grants or consultant fees from Medtronic; others reported none
Li, 2013	For RCT: four lesion-method groups; no sham; abstinence verified via testing	4-group randomized lesion-method trial	NR	Family support encouraged; no structured psychotherapy described	Detoxification prior to surgery; no maintenance meds reported	Neurologic + vital signs intra-op; long-term neuropsych testing	China National “11th Five Years” Scientific Support Plan; Shaanxi Province Government; PLA Health Subdepartment	None reported
Voges, 2013	None (open-label, no sham or control group)	No	NA	Support groups recommended; no psychotherapy provided post-DBS	No anti-craving medications post-DBS	Routine clinical follow-up; reported: one transient hypomania; one electrode displacement discovered via CT	NR	NR
Wang, 2013	None (no control group; pre/post comparison only)	No	NA	NR; follow-up was telephone-based monitoring only	3 participants on methadone maintenance; others drug-free	Postoperative follow-up every 6 months; MRI to rule out complications; no major neurological deficits reported	National Natural Science Foundation of China; Shaanxi Province Science & Technology grants	Authors declare no conflict of interest
Muller, 2016	None (no sham, no randomization)	No	NA	Outpatient psychotherapy offered; some received CBT-like therapy for depression/marital issues	Some received SSRIs (e.g., escitalopram) for depression; anti-craving meds failed prior to DBS	Long-term monitoring; no severe adverse effects except transient hypomania in one patient	Research grants (no specific funder named)	Authors declare no conflict of interest
Goncalves-Ferreira, 2016	Double-blind randomized on/off crossover (2-month alternating periods) + single-blind off-stimulation	Double-blind randomized crossover	Yes double-blind; details NR	Prior psychotherapy (contingency management + family therapy); unclear if continued during DBS	Past methadone for heroin dependence; none reported during DBS	Monitored throughout; transient effects: warmness, sweating, flushing, metallic taste, diminished libido, weight gain; no permanent morbidity	Implant equipment provided by Medtronic	Authors report no biomedical financial interests or conflicts
Chen, 2019	None (open-label, no sham)	No	NA	None reported after surgery	No psychosocial or pharmacologic therapy after surgery; all had prior methadone	Monthly phone follow-ups + in-person urine tests; recorded surgery-related and stimulation-related AEs	Suzhou Sceneray Co. Ltd + National Natural Science Foundation of China	Some authors received consulting fees from Suzhou Sceneray Co. Ltd
Ge, 2019	None (two case reports)	No	NA	NR	NR	Monthly telephone follow-up, urine testing, and clinic visits at 6, 12, 18, 24, and 30 months	National Natural Science Foundation of China; Shaanxi Province Young Stars Project	None declared
Zhang, 2019	None (open-label case report)	No	NA	NR	None reported (no MOUD or other pharmacotherapy)	Monitored for agitation/hypomania; no significant side effects or complications reported	National Key Research & Development Program of China; National Natural Science Foundation of China; Shanghai Science & Technology Committee; Shanghai JiaoTong University; MRC Senior Clinical Fellowship (for Voon)	Sun: research support from PINS & SceneRay; Zhang: honoraria/travel from Medtronic, PINS, SceneRay; others none
Zhang, 2020	Sham stimulation for 9 months (blinded)	No	NA	Weekly psychological counseling	None reported	Urine drug testing weekly; no relapse except one event at 6 months; no surgical complications reported	National Key Research and Development Program of China; Shanghai Jiao Tong University; device donations from SceneRay & PINS	Authors report honoraria/travel from Medtronic, SceneRay, PINS; device donations
Zhu, 2020	None (case report, uncontrolled)	No	NA	NR	NR (no addiction-specific meds reported perioperatively)	Psychiatrist monitored side effects (MOSES scale); no significant adverse events reported	National Key R&D Program of China (2017YFC0803607); Shanghai STCSM (18,410,710,400)	Several authors received honoraria/travel from Medtronic, PINS, SceneRay; devices donated by PINS/SceneRay
Mahoney, 2021	Baseline comparison only (no sham, no randomization)	No	NA	Comprehensive treatment: individual therapy, group therapy, mutual support groups (AA/NA)	Buprenorphine-naloxone (MOUD)	Twice-weekly visits; no serious adverse events reported	National Institute of General Medical Sciences; NIH	NR
Davidson, 2022	Baseline comparison; no sham; no randomization	No (except ON/OFF order for fMRI tasks only)	NA (no randomized clinical trial)	Required ongoing AUD medical follow-up; specifics NR	Some continued previously tried AUD meds; details NR	Monitored at each follow-up; serious AE: 1 IPG infection; others: hypomania, headaches, fatigue	Internal research funds + Harquail Centre for Neuromodulation + philanthropic support to Sunnybrook Foundation	Authors declare no competing interests
Bach, 2023	Sham stimulation (DBS-LATE ON) for first 6 months	Block randomization with variable block sizes (2–4)	Yes investigators and patients blinded; unblinded programmer separate	Standard clinical follow-up; no structured psychotherapy reported	No AUD medications mandated; prior acamprosate/naltrexone failures required	At every visit; independent monitoring board; AEs/SAEs recorded	Deutsche Forschungsgemeinschaft (DFG)	Authors declare no competing interests
Rezai, 2023	None (open-label, single-arm)	No	NA	Standard addiction treatment programs; behavioral therapy; peer recovery support (for abstinent participants)	MOUD (buprenorphine) for all participants before and during study	Monitored throughout; no SAEs or device-related AEs reported	NIDA/NIH HEAL Initiative, award UG3 DA047714	Authors Rezai, Finomore, Mahoney filed two patents related to neuromodulation for addiction
Vorspan, 2023	Double-blind SHAM vs. ON-DBS cross-over	Randomized cross-over (method NR)	NR	CBT (individual), social worker support, residential care	Buprenorphine, cyamemazine, oxazepam, paroxetine, diazepam, aripiprazole, topiramate, zopiclone	Monitored throughout; hypomania documented as serious adverse event	Ministère de la Santé (France), PHRC-Régional 2011, AOR11036	Authors report no commercial or financial conflicts
Qiu, 2024	Sham-controlled acute stimulation test included	No	NA	NR (not described during study period)	Previously on methadone; current MOUD status during study NR	Wound infection monitoring, stimulation side-effects, craving changes; no new adverse events reported	NIH grants, Foundation for OCD Research, Pritzker Consortium, Wellcome Trust, Bayshore Global Management, New Venture Fund	Multiple COIs listed (consulting, patents, equity) for several authors
Lipsman 2025	None (open-label pilot)	No	NA	NR	NR	Safety metrics recorded	Harquail Centre for Neuromodulation + philanthropic support to Sunnybrook Foundation	No significant relationships

*NR* not reported, *NA* not applicable. All values are extracted as reported in the source studies

**Table 5:** Included preclinical studies of neuromodulation in substance use models

Study	Species	Strain	Sex	Sample size/group	Substance model	Paradigm
Henderson, 2010	Rat	Alcohol-preferring (P) rats	Male	Study I: 9 rats (within-subject crossover). Study II: DBS = 6; Sham = 5	Ethanol (10% alcohol)	Voluntary alcohol drinking; ADE (alcohol deprivation effect) model
Rouaud, 2010	Rat	Long-Evans	Male	Varies by experiment: Food FR1, OFF *n* = 8/ON *n* = 16; Cocaine FR1, OFF *n* = 12/ON *n* = 16; PR food, OFF *n* = 8/ON *n* = 14; PR cocaine, OFF *n* = 9/ON *n* = 9; CPP food, OFF *n* = 7/ON *n* = 13; CPP cocaine, OFF *n* = 7/ON *n* = 9	Cocaine (i.v. SA; 250 μg/infusion); sucrose pellets for natural reward comparison	FR1 SA; PR; CPP
Vassoler, 2008	Rat	Sprague–Dawley	Male	Varies by experiment: 7–12 per treatment in main reinstatement experiment; 8 per dorsal striatum DBS experiment; 8–9 in food reinstatement experiment	Cocaine	SA → extinction → cocaine-primed reinstatement
Guo, 2013	Rat	Sprague–Dawley	Male	Experiment 1: *n* = 6 per group (one group had *n* = 5 after exclusion); Experiment 2: *n* = 6 per group (one group *n* = 5 after exclusion)	Heroin (diacetylmorphine HCl)	SA → abstinence → cue-induced reinstatement; SA → abstinence → heroin-induced reinstatement
Ma, 2013	Rat	Sprague–Dawley	Male	Control: 5; Sham DBS: 10; DBS: 10	Morphine	CPP, withdrawal scoring, reinstatement test, Morris water maze
Vassoler, 2013	Rat	Sprague–Dawley	Male	Typically 5–12 per condition depending on experiment (e.g., DBS shell *n* = 5; core *n* = 8; pharmacology *n* = 7–12)	Cocaine	SA → extinction → cocaine-primed reinstatement
Wilden, 2014, Experiment 1 (Pharmacological silencing)	Rat	Alcohol-preferring	Female	8 (Bac-Mus) and 10 (aCSF)	Ethanol (15% EtOH)	Operant SA (FR1 → FR3 → FR5)
Wilden, 2014, Experiment 2 (DBS)	Rat	Alcohol-preferring	Female	3 rats (100 μA), 4 rats (200 μA)	Ethanol (15% EtOH)	Free-choice limited-access drinking (1 h/day)
Evans, 2015	Rat	Long-Evans	Male	20 implanted → 11 completed training → 8 included in final analyses	None (ICSS reward model; no drugs administered)	ICSS; operant conditioning; preference testing
Guercio, 2015	Rat	Sprague–Dawley	Male	*n* = 6 for cocaine DBS experiment; *n* = 6 for sucrose DBS experiment	Cocaine; sucrose (natural reward)	SA → extinction → cue-induced reinstatement
Hamilton, 2015	Rat	Long-Evans	Male	After exclusions: SA-Sham *n* = 6; Co-Sham *n* = 7; Co-LF *n* = 9; Co-HF *n* = 8	Cocaine	SA + reinstatement/relapse testing
Hadar, 2016	Rat	Wistar	Male	Chronic DBS: *n* = 10–15 per group; Acute DBS: *n* = 6–9 per group; fMRI: *n* = 6; microdialysis: *n* = 6–7	Ethanol	Alcohol Deprivation Effect (ADE) relapse model (relapse-like drinking)
Martinez-Rivera, 2016	Rat	Sprague–Dawley	Male	Varies: HF dorsal-VS *n* = 11; HF ventral-VS *n* = 9; sham *n* = 23; LF DBS *n* = 14; LF sham *n* = 15; c-Fos: sham *n* = 6, HF *n* = 4, LF *n* = 5	Morphine	CPP + extinction
Batra, 2017	Rat	Wistar	Male	DBS: *n* = 10; Sham: *n* = 8	Methamphetamine	IV SA, extinction, relapse (pavlovian spontaneous recovery)
Guercio, 2020	Rat	Sprague–Dawley	Male	Varies by experiment: IL: 8–9; PL: 7–8; ACC: 7–8; BLA: 7; vHipp: 6–8	Cocaine; sucrose (as natural reward control)	Cocaine SA → extinction → reinstatement (drug-primed); sucrose SA → extinction → reinstatement
Yang, 2020	Rat	Sprague–Dawley	Male	10 per subgroup (20 per major group; 4 subgroups total)	Morphine	CPP, extinction, reinstatement
Kallupi, 2022	Rat	Sprague–Dawley (Charles River)	Male	Sham *n* = 6; DBS *n* = 7 (after excluding 1 for misplaced electrode)	Cocaine	SA (extended access), irritability-like behavior, locomotor activity
Esfahlani, 2023	Rat	Wistar	Male	8 rats per group	Morphine (saline control also used)	CPP; withdrawal relapse and extinction tests also performed
Eskandari, 2023	Rat	Wistar	Male	4–7 rats per group (SHAM *n* = 4, LFS *n* = 6, HFS *n* = 7; misplacements excluded)	Methamphetamine	CPP, extinction, reinstatement
Fatemizadeh, 2025	Rat	Wistar	Male	Saline: 6; Sham-DBS: 14; DBS-150C: 5; DBS-150E: 5; DBS-200C: 10; DBS-200E: 10	Morphine	CPP, extinction, reinstatement; plus NOR and open-field locomotion tests
Gong, 2025	Mouse	C57BL/6	Male	Varies by experiment (ranges 3–14 per group)	Morphine	CPP, reinstatement; fiber photometry; chemogenetics
Lloret-Torres, 2025	Rat	Sprague–Dawley	Male	Cohort 1: *n* = 12 Sham-DBS, *n* = 12 DBS; Cohort 2: *n* = 3 per group	Morphine	CPP, extinction, reinstatement
Vignal, 2025	Rat	Lister-Hooded	Male	Experiment 1: ON *n* = 13, OFF *n* = 15 (after exclusions); Experiment 2: ON *n* = 12, OFF *n* = 12 (after exclusions)	Ethanol (10% for SA; 20% for IAA escalation)	Operant SA (FR1); intermittent access (IAA) escalation model

*NA* not applicable, *NR* not reported. All values are extracted as reported in the source studies

*NAc* nucleus accumbens

**Table 6:** Preclinical targets and stimulation protocols

Study	Surgery type	Target	Laterality	Coordinates (AP/ML/DV)	Stimulation parameters	Session schedule	Closed-loop/adaptive	Pharmacology	Verification	Complications
Henderson, 2010	DBS electrode implantation	NAc (shell region targeted, but stimulation spread likely beyond shell)	Bilateral	AP + 1 mm; ML ± 3 mm; DV −8 mm from brain surface; Paxinos & Watson atlas	Mode: Continuous during treatment periods (1-h on/off cycles in Study I; continuous 24-h in Study II); f: 140–150 Hz; PW: 60 µs; Amp: 200 µA (current); Duty: NR; inferred continuous during “on” periods → NR	Duration: Study I: 60-min DBS or sham periods; Study II: 24-h continuous DBS; Sessions: Study I: 2 days × (1-h DBS + 1-h sham). Study II: 1 × 24-h session	No (open-loop)	No additional drugs; only ethanol exposure	Histology with cresyl violet; electrode tracks identified	Tissue damage at electrode tip noted; no behavioral complications reported
Rouaud, 2010	DBS electrodes implanted bilaterally	STN	Bilateral	AP + 3.7 mm, ML ± 2.4 mm, DV −8.3 mm from skull; Paxinos & Watson atlas	Mode: Continuous during behavioral sessions; f: 130 Hz; PW: 60 µs; Amp: 50–130 µA (set individually just below hyperkinetic threshold); Duty: Continuous (no cycling reported)	Duration: PR cocaine sessions averaged 160 ± 6 min; others NR; Sessions: PR: 10 sessions for reversibility test; others NR but implied daily sessions	No (open-loop)	Cocaine (i.v. SA), cocaine 10 mg/kg i.p. for CPP; sucrose pellets for natural reward	Histology (cresyl violet staining; electrode track confirmation)	NR
Vassoler, 2008	DBS electrode implantation + jugular catheterization	NAc shell (primary); dorsal striatum (control site)	Bilateral	NAc shell: AP + 1.0, ML ± 3.0, DV −7.3 mm (Paxinos & Watson, 1997); Dorsal striatum: AP + 1.0, ML ± 4.0, DV −5.0 mm	Mode: Continuous DBS during entire reinstatement session; f: 160 Hz; PW: 60 µs per phase (biphasic symmetrical pulses); Amp: 150 µA (effective intensity); tested 70–150 µA; Duty: Continuous (100%)	Duration: 120 min (2 h) reinstatement sessions; Sessions: Multiple reinstatement sessions across 16-day reinstatement phase; exact per-animal count varies due to counterbalancing	No (open-loop)	Cocaine priming injections (0, 5, 10, 20 mg/kg i.p.)	Histology (coronal sections, electrode placement confirmation)	NR
Guo, 2013	DBS electrodes implanted; also IV catheter implantation	NAc core	Bilateral (Experiment 1); unilateral left or right (Experiment 2)	AP + 1.6 mm; ML ± 1.8 mm; DV −7.2 mm from bregma; Paxinos & Watson (1997) atlas	Mode: Continuous stimulation during 1-h daily sessions; f: 130 Hz; PW: 100 µs; Amp: 0–150 µA (0 µA, 75 µA, 150 µA groups); Duty: Continuous biphasic pulses (no intermittent duty cycle reported)	Duration: 60 min per day; Sessions: 7 days of DBS before cue-induced reinstatement; 7 additional days before heroin-induced reinstatement	No (open-loop)	Heroin SA; heroin priming injection (250 µg/kg SC) for reinstatement test	Histology with cresyl violet; electrode tip localization; exclusion of off-target placements	NR
Ma, 2013	DBS electrode implantation	NAc shell	Bilateral	AP + 2.0–2.2 mm; ML 1.2–1.5 mm; DV −6.5 to −7.5 mm; relative to bregma; Paxinos & Watson atlas	Mode: Intermittent (5 sessions/day; each 60 min with 15-min intervals); f: 130 Hz; PW: 60 µs; Amp: 2 V; Duty: NR; stimulation delivered in discrete 60-min trains	Duration: 60 min per train; Sessions: 5 trains/day × 30 days = 150 stimulation sessions	No (open-loop)	Morphine (conditioning, reinstatement), naloxone (withdrawal induction)	Histological verification implied (electrode placement described), but explicit histology results not detailed in text provided	NR
Vassoler, 2013	DBS electrode implantation or guide cannula implantation	NAc shell; NAc core; infralimbic cortex; prelimbic cortex; ACC	Bilateral electrodes or bilateral microinjections	Accumbens shell (electrodes): AP + 1.0, ML 3.0, DV 7.3, 17° angle. Accumbens core: AP + 1.0, ML 3.0, DV 7.07, 8.13° angle. Cannulae: Shell AP + 1.0, ML 1.0, DV 5.0; Core AP + 1.0, ML 2.5, DV 5.0; IL AP + 2.5, ML 0.5, DV 3.0; PL AP + 2.5, ML 0.5, DV 2.0; Cg1 AP + 2.5, ML 0.5, DV 1.0 (Paxinos & Watson, 1997)	Mode: Continuous during 2-h reinstatement session; f: 160 Hz; PW: 60 µs (biphasic symmetrical pulses); Amp: 150 µA (0 µA for control); Duty: Continuous (100%)	Duration: 120 min (2 h) reinstatement session; Sessions: Multiple reinstatement sessions over 16 days; DBS delivered on specific reinstatement test days (counterbalanced)	No (open-loop)	Baclofen/muscimol; lidocaine; bicuculline + saclofen; cocaine priming injection	Histology: electrode/cannula placement verification; c-Fos immunohistochemistry	NR
Wilden, 2014, Experiment 1 (Pharmacological Silencing)	Bilateral guide cannula implantation	NAc shell	Bilateral	AP + 1.6, ML ± 2.4, DV −7.5 mm (10° angle), Paxinos & Watson atlas	NA	Duration: 60 min; Sessions: 7 acquisition injections + 5 maintenance injections	No	Baclofen + muscimol (0.3 nmol + 0.03 nmol)	Histology with bromophenol blue injection	NR
Wilden, 2014, Experiment 2 (DBS)	Unilateral DBS electrode implantation	NAc shell	Unilateral (left)	AP + 1.6, ML + 2.4, DV −8.5 mm (10° angle), Paxinos & Watson atlas	Mode: Continuous during 1-h session (plus 5-min pre-stimulation); f: 150 Hz; PW: 100 µs; Amp: 100 or 200 µA; Duty: Continuous (no cycling reported)	Duration: 60 min (plus 5-min pre-stimulation); Sessions: 5 days (100 µA) or 6 days (200 µA) of DBS + 3 post-DBS days	No	No	Histology; impedance testing for electrode function	NR
Evans, 2015	DBS electrode implantation (no stimulation during surgery; stimulation used behaviorally)	NAc shell	Unilateral (left)	AP + 1.2 mm; ML + 3.0 mm at 17° medial angle; DV −7.0 mm (relative to bregma; Paxinos & Watson atlas)	Mode: Multiple paradigms: tonic, intermittent tonic, burst; f: 130–500 Hz depending on paradigm; PW: 1000 µs (reduced to 500 µs in animals showing stereotypy); Amp: 200–400 µA (constant current); Duty: Paradigm-dependent (e.g., IT-50/0/0 = 50 pulses every 3 s; burst patterns vary)	Duration: Preference tests: 60-s epochs repeated; total session 15–30 min; Sessions: Up to 5 per week; total sessions per rat 37–103 depending on training progression	No (all stimulation open-loop, experimenter-programmed)	No	Histology with cresyl violet; electrode placement confirmed; misplacements excluded	Stereotypic movements in some rats; seizure-like behaviors at high pulse width; these animals excluded
Guercio, 2015	DBS electrodes + jugular catheter implantation	NAc shell	Bilateral	AP + 1.0 mm; ML ± 3.0 mm; DV −7.3 mm; 17° angle; relative to bregma (Paxinos & Watson)	Mode: Continuous during entire reinstatement session; f: 160 Hz; PW: 60 µs (biphasic symmetrical pulses); Amp: 150 µA (0 µA for control); Duty: Continuous (no cycling reported)	Duration: 120 min (cocaine reinstatement); 60 min (sucrose reinstatement); Sessions: Two reinstatement sessions per animal (counterbalanced)	No (open-loop)	Cocaine (0.254 mg/infusion); sucrose pellets; ketamine/xylazine anesthesia; heparinized saline + antibiotic for catheter maintenance	Histology (coronal sections, electrode placement verification)	NR; animals with misplaced electrodes excluded
Hamilton, 2015	DBS electrode implantation + jugular catheter	NAc, central region; placements in both core and shell (mixed)	Unilateral (right)	AP + 1.3 mm; ML −1.4 mm; DV −7.1 mm from brain surface; Paxinos & Watson (2006) atlas	Mode: Continuous biphasic stimulation; f: LF = 20 Hz; HF = 160 Hz; PW: 60–100 µs (HF typically 60 µs; LF based on prior work ~ 100 µs; paper references both); Amp: Individually adjusted; target ~ 150 µA (range 50–200 µA); Duty: NR (continuous stimulation for 30 min)	Duration: 30 min/day; Sessions: 14 consecutive days	No (open-loop)	Cocaine SA (0.5 mg/kg/infusion) and cocaine priming (5 mg/kg i.p.)	Histology (cresyl violet)	4 animals excluded due to incorrect electrode placement; no behavioral/motor complications reported
Hadar, 2016	DBS electrode implantation	NAc shell; CPu and IL cortex in comparison experiments	Bilateral	NAc shell: AP + 1.6 mm, ML ± 0.8 mm, DV −7.6 mm from dura (Paxinos & Watson atlas)	Mode: Chronic-continuous DBS (main experiment); acute 24-h DBS (replication experiment); f: 130 Hz; PW: 90 µs; Amp: 5.25 V (constant-voltage mode); Duty: Continuous (no cycling reported)	Duration: Chronic: 3 days pre-relapse + 4 days post-relapse (continuous); Acute: 24 h; Sessions: Chronic: 1 continuous stimulation block; Acute: 1 session	No (open-loop)	Ethanol injections during microdialysis (0.5, 1.0, 1.5 g/kg)	Electrode localization verified histologically; fMRI activation mapping also performed	NR
Martinez-Rivera, 2016	DBS electrode implantation	Dorsal-VS or ventral-VS	Bilateral	Dorsal-VS: AP + 1.2, ML ± 3.5, DV −6.5 mm; Ventral-VS: AP + 1.2, ML ± 3.5, DV −8.0 mm (Paxinos & Watson atlas)	Mode: Continuous 60-min sessions (20 min home cage → 20 min CPP → 20 min home cage); f: High frequency: 130 Hz; Low frequency: 20 Hz; PW: 0.1 ms = 100 µs; Amp: 100–200 µA (constant current); Duty: Continuous (no cycling reported)	Duration: 60 min per day; Sessions: HF: 11 sessions; LF: 9 sessions; c-Fos: 6 sessions	No (open-loop)	Morphine 5 mg/kg SC; no other pharmacology during DBS	Histology (cresyl violet) + electrode placement confirmation	NR
Batra, 2017	DBS electrode implantation	NAc shell	Bilateral	1.8 mm AP from bregma, ± 2.4 mm ML, −8.5 mm DV, 10° angle (Paxinos atlas)	Mode: Intermittent (3 h/day, separate environment); f: 130 Hz; PW: 60 µs; Amp: 200 µA (constant current); Duty: Continuous during 3 h session (within each day)	Duration: 180 min stimulation + 120 min operant session; Sessions: 5 consecutive days	No (open-loop)	Methamphetamine IV SA only	Histology (electrode tip lesioning, brain slicing, atlas confirmation)	NR
Guercio, 2020	DBS electrode implantation (bipolar stainless steel electrodes)	Infralimbic cortex (IL); prelimbic cortex (PL); ACC; basolateral amygdala (BLA); ventral hippocampus (vHipp)	Bilateral (coordinates provided for ± ML placements)	IL: + 2.5 AP, ± 2.0 ML, −5.39 DV, 21.78° angle; PL: + 2.5 AP, ± 2.0 ML, −4.2 DV, 19.5° angle; ACC: + 2.5 AP, ± 2.0 ML, −3.0 DV, 27.8° angle; BLA: −2.8 AP, ± 5.0 ML, −8.5 DV; vHipp: −5.5 AP, ± 5.0 ML, −6.5 DV (Paxinos & Watson, 1997)	Mode: Continuous stimulation during entire reinstatement session; f: 160 Hz; PW: 60 µs (biphasic symmetrical pulses); Amp: 150 µA (0 µA for control); Duty: Continuous (100%)	Duration: 120 min (2-h reinstatement sessions); Sessions: Two reinstatement test days per animal (counterbalanced 0 vs 150 µA)	No (open-loop)	Cocaine 10 mg/kg i.p. for reinstatement; sucrose pellets for natural reward reinstatement. No pharmacological manipulation of DBS targets	Histological verification of electrode placement (100 µm coronal sections)	NR
Yang, 2020	DBS electrode implantation (bipolar stainless steel)	NAc, core region targeted via coordinates	Bilateral	AP + 1.5 mm, ML ± 2.0 mm, DV −6.4 mm (Rat Brain Atlas 5th ed., 2004)	Mode: Daily sessions; appears intermittent (30 min/day); f: 120 Hz; PW: 60 µs (reported as 60 ms in text but consistent with typical DBS parameters; paper states “60 ms”); Amp: 0.3 mA; Duty: NR	Duration: 30 min per session; Sessions: Extinction group: 9 sessions; post-extinction group: 7 sessions	No (open-loop)	Morphine (10 mg/kg conditioning; 3 mg/kg reinstatement)	Histology (H&E staining) to confirm electrode placement	NR
Kallupi, 2022	DBS electrode implantation (bipolar stainless steel)	NAc shell	Bilateral	AP + 1.0 mm; ML ± 3.0 mm; DV −7.0 mm from dura; 17° angle toward midline; Paxinos & Watson atlas	Mode: High-frequency monophasic stimulation; intermittent (ON/OFF sessions); f: 130 Hz; PW: 60 µs; Amp: 150 µA constant current (stimulator set to 1.5 V, isolator to 1 mA); Duty: ON for 2 h (30 min pre-session + 90 min during SA), then OFF for remainder of 6-h session	Duration: 120 min stimulation per ON day; SA sessions 360 min total; Sessions: 4 DBS test days (2 ON, 2 OFF)	No (open-loop)	Cocaine SA; cocaine 10 mg/kg i.p. challenge	Histological verification of electrode placement (fresh-frozen sections)	1 rat excluded due to misplaced electrodes
Esfahlani, 2023	DBS electrode implantation	mPFC stimulation; recording electrode in NAc	NR	AC: AP −4 mm, ML ± 1.6 mm, DV −8.4 mm (Paxinos atlas)	Mode: Intermittent (15 min stimulation, 45 min interval, repeated for 3 h); f: 130 Hz; PW: NR; Amp: 0.2–0.5 mA; Duty: NR (pattern described above)	Duration: 15 min per stimulation bout; Sessions: 3 h total per conditioning day (multiple bouts)	No (open-loop)	Morphine IP injections; saline controls	Electrophysiological single-unit recording; histological verification NR	NR
Eskandari, 2023	DBS electrode implantation	NAc shell	Bilateral	AP + 1.5 mm, ML ± 1.0 mm, DV −7.7 mm (relative to Bregma; Paxinos & Watson atlas)	Mode: Continuous (30 min sessions); f: LFS: 10 Hz; HFS: 130 Hz; PW: 100 µs; Amp: 150–200 µA (stepwise increments); Duty: NR (continuous square pulses)	Duration: 30 min; Sessions: Daily during extinction (10 days)	No (open-loop)	Methamphetamine injections (conditioning: 1 mg/kg; reinstatement: 0.25 mg/kg); saline controls	Histology (coronal slices, electrode placement confirmed)	Misplacements (*n* = 7) excluded; no other complications reported
Fatemizadeh, 2025	DBS electrode implantation	ACC	Bilateral	AP + 2.0 mm, ML ± 0.6 mm, DV −3.0 mm (relative to bregma; atlas NR but likely Paxinos & Watson)	Mode: Continuous during sessions (40 min during conditioning; 20 min during extinction); f: 130 Hz; PW: 100 µs; Amp: 150 µA or 200 µA (current-based stimulator); Duty: NR (continuous stimulation implied)	Duration: Conditioning: 40 min; Extinction: 20 min; Sessions: Conditioning: 3 days; Extinction: until extinction (2–11 days depending on group); Reinstatement: 1 session	No (open-loop)	Morphine (3, 5, 7 mg/kg conditioning; 2 mg/kg priming)	Histology (coronal sections showing electrode tracks)	NR
Gong, 2025	DBS electrode implantation; viral injections; fiber photometry cannula	NAc; mPFC: PrL + IL	Bilateral NAc electrodes; bilateral mPFC viral injections	NAc: AP + 1.40, ML ± 0.65, DV −4.2 mm; mPFC: AP + 1.95, ML ± 0.4, DV −1.8 mm (Paxinos & Watson)	Mode: Continuous DBS (2 h/day); f: 130 Hz; PW: 90 µs; Amp: 50 µA; Duty: Continuous (no cycling reported)	Duration: 120 min/day (2 h); Sessions: 14 consecutive days (main CPP experiment); 4 days for c-Fos mapping	No (open-loop)	Morphine; CNO for DREADDs; D1/D2 antagonists in fiber photometry experiment	Histology (c-Fos, mCherry, GFP), immunohistochemistry, fiber placement confirmation	NR
Lloret-Torres, 2025	DBS electrode implantation	VS/NAc	Bilateral	AP + 1.2 mm; ML ± 3.3 mm; DV − 6.5 mm; 10° angle; Paxinos & Watson atlas	Mode: Continuous during 60-min extinction block (3 × 20-min segments); f: 20 Hz (low-frequency DBS); PW: 0.1 ms = 100 µs; Amp: 100–200 µA (constant current); Duty: Continuous stimulation (no cycling reported)	Duration: 60 min per extinction day (20 min home cage → 20 min CPP chamber → 20 min home cage); Sessions: Cohort 1: ~ 10 days DBS vs ~ 45 days Sham to reach extinction; Cohort 2: single 60-min DBS session	No (open-loop)	Morphine 5 mg/kg s.c. for conditioning and reinstatement	Histology (cresyl violet), fluorescence microscopy for tracer placement, IHC for BDNF	NR (animals with misplaced electrodes excluded)
Vignal, 2025	Bilateral electrode implantation for DBS	STN	Bilateral	AP −3.7 mm, ML ± 2.4 mm, DV −8.35 mm from skull; Paxinos & Watson (2014) atlas	Mode: Continuous during 1-h sessions; f: 130 Hz; PW: 80 µs; Amp: 50–150 µA (set just below threshold for abnormal movements); Duty: Continuous (no cycling reported)	Duration: 60 min per SA session; Sessions: Experiment 1: 5 alone + 5 with peer; Experiment 2: 5 alone + 5 with peer; IAA: 15 sessions	No (open-loop)	Anesthesia (ketamine, medetomidine), antibiotics (amoxicillin), analgesia (meloxicam), reversal (atipamezole)	Histology with cresyl violet; misplacements excluded	NR; animals with mislocated electrodes excluded (*n* = 2)

*NR* not reported, *NA* not applicable. All values are extracted as reported in the source studies

*ACC* Anterior cingulate cortex, *ALIC* anterior limb of the internal capsule, *AUD* alcohol use disorder, *CPP* conditioned place preference, *DBS* deep brain stimulation, *ICSS* intracranial self-stimulation, *mPFC* medial prefrontal cortex, *NAc* nucleus accumbens, *OUD* opioid use disorder, *PR* progressive ratio, *SA* self-administration, *STN* subthalamic nucleus, *VC* ventral capsule, *VS* ventral striatum

### Clinical DBS evidence

#### Alcohol use disorder

##### Randomized evidence

The most rigorous clinical evidence comes from a randomized delayed-activation design evaluating bilateral NAc DBS in severe, treatment-refractory alcohol use disorder [1]. Across the full cohort (*n* = 12), abstinent days increased from 27.6% ± 11.6 at baseline to 56.0% ± 31.1 at 6 months (*p* = 0.014) and to 74.2% ± 31.3 at 18 months (*p*= 0.004) [1]. Heavy drinking days decreased from 69.6% ± 12.6 to 47.4% ± 27.6 at 6 months (*p* = 0.020) and to 35.5% ± 29.9 at 18 months (*p*= 0.031) [1]. Mean daily alcohol intake dropped from 207.4 ± 162.2 g/day to 85.3 ± 69.7 g/day at 6 months (*p* = 0.014) and 62.7 ± 72.5 g/day at 18 months (*p*= 0.037) [1]. Craving improved significantly: OCDS declined from 21.0 ± 7.0 to 10.5 ± 7.2 at 6 months (*p* = 0.014) and 5.9 ± 6.4 at 18 months (*p*= 0.008) [1]. Blinded-phase comparisons favored earlier stimulation for abstinent days and heavy drinking days (*p* = 0.048), and also showed lower AUQ craving (*p* = 0.020) and reduced anhedonia (SHAPS *p* = 0.028; social anhedonia *p*= 0.008) [1]. Safety reporting noted 38 adverse events in the first 6 months, including alcohol-relapse related hospitalizations classified as serious adverse events [1].

##### Prospective pilot trial evidence

In a phase I DBS trial for severe alcohol use disorder targeting the NAc, Davidson and colleagues reported clinically meaningful reductions in alcohol consumption and craving at 12 months [5]. Self-reported standard drinks/day decreased from 10.4 ± 4.0 pre-DBS to 2.7 ± 3.0 at 12 months (paired Wilcoxon *p*< 0.05) [5]. Alcohol Use Disorders Identification Test scores declined from 35.7 ± 3 to 11.0 ± 12 (*p* < 0.05), craving (ACQ) from 58.3 ± 15 to 41.8 ± 17 (*p*< 0.05), and dependence severity (ADS) from 26.0 ± 7 to 9.0 ± 11.0 (not statistically significant) [5]. OCDS dropped from 28.7 ± 5 to 8.3 ± 8 (*p* < 0.05), with anxiety reductions on BAI (20.3 ± 9 to 9.3 ± 12; *p*< 0.05). The study also reported improvements in liver enzymes with strong model effects (ALT β = 3.40, CI 0.80–6.01, p-FDR < 0.01; AST β = 2.38, CI 0.98–3.77, p-FDR = 0.01) [5]. These clinical improvements were accompanied by neuroimaging correlates including reduced NAc metabolism on FDG-PET and changes in NAc connectivity/cue reactivity on fMRI [5, 33].

##### Case series and long-term outcomes

Multiple case reports and small series suggest that some individuals experience sustained reduction in craving and prolonged abstinence after NAc DBS. Kuhn et al. described one patient with severe alcohol dependence who transitioned from heavy daily consumption to near abstinence, with complete cessation after one year; carbohydrate-deficient transferrin normalized from 7.8% to 1.9% and craving/dependence scores reportedly fell below pathological thresholds [31]. Muller et al. reported early experience treating three alcohol-dependent patients with NAc DBS; two remained abstinent and one showed marked reduction [41]. Quantitatively, AUQ dropped sharply (e.g., from 53 to 8 at 6–12 months in one case) and OCDS obsession/compulsion subscores fell to 0 at follow-up in two out of three patients [41]. Longer-term follow-up of five patients treated with NAc DBS suggested durable craving suppression and functional recovery in a subset: all reported absence of craving, two remained abstinent for years, and three reduced drinking substantially with follow-up up to eight years [42]. Voges et al. reported a five-patient alcohol NAc DBS series with follow-up extending beyond three years in several cases; standardized measures again showed large reductions in craving (AUQ) and OCDS across cases, and durable abstinence in some individuals [56]. Mechanistic imaging work in an abstinent alcohol-dependent DBS patient demonstrated attenuated cue-related activation patterns and highlighted stimulation-related neuropsychiatric effects such as transient hypomania responsive to parameter adjustment [23].

#### Opioid and heroin use disorders

##### DBS cohort and pilot trial evidence

A pilot cohort study of DBS in heroin-use disorder targeting the NAc and ALIC reported sustained abstinence in a subset and large improvements in craving and quality of life among non-relapsed participants [4]. Five of eight patients remained abstinent for > 3 years, while two relapsed within the first year (one patient lost to follow up at 3 months) [4]. Among non-relapsed patients, craving visual analog scale (VAS) decreased from 7.6 ± 1.34 baseline to 1.4 ± 1.10 at 3 months and 0.8 ± 0.84 at 24 months (*p*< 0.001) [4]. Depression and obsessive–compulsive symptom measures improved (Hamilton depression rating scale 12.4 ± 6.23 to 3.4 ± 2.19 at 24 months; *p* = 0.001; Yale-Brown Obsessive Compulsive Scale [OCS]; 21.4 ± 10.92 to 3.8 ± 2.17; *p*< 0.001) [4]. Quality-of-life summary scores increased from 504.9 ± 145.1 to 704.0 ± 73.6 at 24 months (*p*= 0.001) [4].

##### Single-case reports

Several single-case reports support possible benefits. Valencia-Alfonso et al. described heroin addiction treated with bilateral NAc DBS, reporting substantial reductions in daily heroin intake and craving depending on active contact selection; craving scores (Desire/Interest scale) dropped from 33 pre-DBS to 18 and heroin intake dropped from 0.50 g/day to 0.10 g/day under dorsal contact stimulation, whereas alternative contacts worsened craving and intake [52]. Zhou et al. reported long-term (6-year follow-up) abstinence in a heroin-dependent patient treated with NAc DBS, noting drug-free status during stimulation and continued abstinence (3.5 years) after stimulation discontinuation [68]. Zhang described a patient who maintained abstinence through an extended (9-month) blinded sham-stimulation period and subsequently underwent safe DBS explantation 2 years after successful treatment, highlighting a rarely discussed endpoint: elective device removal after sustained recovery [66]. A combined approach (NAc DBS + anterior capsulotomy) was reported in a polysubstance case with improvements across craving, mood, anxiety, sleep, and quality of life over 12 months; craving for opioids reduced from VAS 6 to 0 by 3 months and remained low, with large improvements in depression/anxiety scores over time (Hamilton Depression Rating Scale baseline 26 to 12 at 12 months, Hamilton Anxiety Rating Scale baseline 29 to 11 at 12 months) [69].

##### Contemporary controlled feasibility trial evidence

Mahoney et al. reported an IRB/FDA-regulated single-participant trial of NAc/VC DBS for severe opioid and benzodiazepine use disorder [36]. The participant remained abstinent at both 12 weeks and 12 months [36]. Benzodiazepine craving decreased from 53.4 ± 29.5 pre-DBS to 1.0 ± 2.2 at 12 weeks (*p*< 0.001) with similar benefit at 12 months [36]. Heart rate variability increased from 19.2 ± 8.2 to 56.0 ± 11.7 (*p*< 0.001) [36]. Behavioral risk task performance improved with a trend toward significance (*p*= 0.06) [36].

Rezai et al. reported a multi-participant feasibility study in severe polysubstance use disorder using DBS targeting reward circuitry; four male participants were enrolled, with two maintaining complete abstinence for > 1150 and > 520 days [45]. Statistically significant craving reductions were observed in multiple participants across substances (e.g., participant 2: opioid craving *F* = 5.41, *p* = 0.036; benzodiazepine craving *F* = 16.91, *p* = 0.001; cannabis craving *F* = 8.77, *p* = 0.010; participant 3: heroin craving *F* = 30.03, *p* < 0.001; benzodiazepine craving *F* = 54.07, *p* < 0.001; cannabis craving *F* = 62.30, *p*< 0.001) [45]. Not all participants benefited; one showed nonsignificant changes (e.g., heroin craving *p*= 0.332) [45]. Qiu et al. reported a biomarker-guided optimization study in opioid use disorder demonstrating that personalized stimulation parameters correlated with > 50% reduction in cue-induced opioid craving (mean 6.93 vs 3.09; *p* = 4.32 × 10⁻⁷) and physiologic modulation of low-frequency activity (*p*= 0.039) [44].

#### Stimulant use disorders (cocaine and methamphetamine)

##### Cocaine

Goncalves-Ferreira et al. described DBS in a patient with severe refractory cocaine dependence, reporting improvement across objective and subjective measures [15]. Percentage of weeks free of cocaine increased from 40.9% pre-DBS to 80.5% at 6 months and 68% at 24 months; craving VAS dropped from 3.4 to 1.0 at 6 months and 0 at 24 months; negative urine screens rose from 12.5% to 66.7% at 6 months and 56.5% at 24 months [15]. Vorspan et al. reported STN DBS in a patient with crack cocaine dependence in a double-blind crossover design; STN stimulation did not clearly reduce crack craving or use and was associated with psychiatric destabilization in a context of cocaine intake [58].

##### Methamphetamine

Two case-based DBS experiences were reported for methamphetamine use disorder. Ge et al. described two methamphetamine-dependent patients treated with NAc DBS; one remained drug-free 1.5 years with craving VAS reduction from 5 to 0 and improved psychoticism scores (SCL-90 psychoticism 3.0 to 1.3), while a second patient relapsed when stimulation amplitude was reduced because of adverse reactions [13]. In this second patient, the right ventral DBS electrode contact deviated from the NAc region [13]. Zhang et al. reported a case of NAc plus VC DBS in methamphetamine dependence with improvements in craving and markers of dopamine system recovery: craving VAS decreased from 8 to 0 and OCS scores reduced from 29 to 11 at one year, while dopamine transporter binding increased in the caudate (+ 20.5%) and putamen (+ 25.6%) [67].

### Clinical lesion/Ablation evidence

Lesion approaches are summarized separately from DBS because they differ fundamentally in reversibility, adjustability, safety profile, and ethical threshold. Whereas DBS permits post-operative programming changes and discontinuation of stimulation, lesion approaches produce permanent structural disruption of the targeted tissue or fiber pathways. Therefore, ablation findings are interpreted here as historical and mechanistic evidence for the relevance of ventral striatal circuitry, not as directly interchangeable evidence for DBS efficacy.

#### Ablation for alcohol dependence

Wu et al. reported stereotactic NAc ablation in 12 alcohol-dependent patients with follow-up 6–27 months; relapse occurred in 3 of 12, and standardized scales improved significantly at 6 months: Severity of alcohol dependence scale dropped from 39.08 ± 3.78 to 9.25 ± 10.52 (*p* < 0.01), drinking days in the last month from 21.17 ± 3.10 to 3.92 ± 7.61 (*p* < 0.01), and average drinks/day from 9.00 ± 2.22 to 2.25 ± 4.52 (*p*< 0.01) [63]. While these results suggest possible efficacy, interpretation is constrained by design limitations.

#### Ablation and intraoperative stimulation findings relevant to addiction

Ablative procedures targeting the NAc have been reported in multiple series, particularly for opioid dependence. Gao et al. described 28 opiate-dependent patients undergoing NAc ablation with follow-up to 15 months; relapse rates increased over time (7.7% within 1 month, 38.5% between 1 and 6 months, and 57.5% after > 6 months), though major surgical complications were reportedly uncommon [11]. Li et al. reported larger cohorts (*n* = 78) and methodological comparisons, noting a 4-year abstinence rate of 53.8% overall (42/78), with markedly higher abstinence rates in certain targeting/technique groups (75.0% and 76.2% in groups C and D vs. 31.3% and 28.6% in groups A and B; *p*< 0.005) [32]. Neuropsychological adverse effects were reported in 24.3% (18/74) of patients, though most were described as transient; no intracranial hemorrhage or infection was reported in that series [32]. Wang et al. examined reward-domain changes after NAc ablation in heroin-recovered individuals, documenting alterations in appetite/weight and sexual function measures with several statistically significant changes (e.g., increased sexual drive; higher overall satisfaction; and increased appetite and weight) [59]. Fang et al. reported intraoperative electrical stimulation responses in heroin-dependent patients, including autonomic changes and intense euphoria similar to heroin-induced euphoria associated with stimulation of the NAc and amygdala; combined stimulation strongly predicted heroin-like euphoria (OR ~ 5.4, *p*< 0.0001) [9]. These findings show that NAc manipulation can modify affective reward responses and may have unintended motivational consequences.

### Preclinical evidence (Animal Studies)

Across 23 animal studies, DBS effects varied by substance, dependence state, stimulation frequency, timing (acquisition vs. extinction vs. reinstatement), and precise target location (core vs. shell; infralimbic vs. prelimbic vs. ACC; STN vs. striatal targets). Several consistent themes emerged.

#### Cocaine models

Reduction of cocaine seeking and reinstatement was frequently observed with NAc shell DBS and related circuit targets. DBS of the NAc shell was shown to attenuate cocaine priming-induced reinstatement [53] and that shell DBS may act via broader cortical-striatal modulation rather than simple local inactivation [54]. DBS in glutamatergic input regions such as the infralimbic cortex also showed reduced cocaine-primed reinstatement with statistically supported interaction effects (e.g., *F* = 7.876, *p*= 0.0263;) [18]. Chronic unilateral NAc DBS reduced relapse behavior after withdrawal with reductions of 36–48% at peak relapse time points (day 15 post-withdrawal) [22]. However, not all outcomes were uniformly beneficial: in cocaine-dependent extended-access rats, NAc shell DBS slightly increased cocaine self-administration while reducing withdrawal-associated irritability and psychomotor activation, suggesting complex motivational tradeoffs [26].

#### Opioid/Heroin models

Heroin/opioid relapse models showed promising effects of NAc stimulation in some paradigms. Guo et al. demonstrated that bilateral NAc core DBS attenuated cue- and heroin-induced reinstatement, with clear group differences (e.g., heroin-induced reinstatement *F* = 7.74, *p* = 0.001; DBS groups significantly reduced responding vs. controls, *p*< 0.05) [20]. Morphine CPP studies showed that prolonged high-frequency DBS could prevent reinstatement without impairing memory [35] and that ACC DBS inhibited morphine reward and facilitated extinction with intensity-dependent effects [10]. Low-frequency VC/VS DBS appears to strengthen extinction memory and modulate brain-derived neurotrophic factor signaling (133% increase) in corticomesolimbic regions [34].

#### Methamphetamine models

Methamphetamine models similarly supported relapse-reducing potential depending on stimulation pattern. Eskandari et al. reported that both high- and low-frequency (130 Hz and 10 Hz) NAc shell DBS reduced extinction time and prevented methamphetamine-primed reinstatement under specific timing conditions, with synchronous protocols often outperforming nonsynchronous stimulation [7]. Batra et al. reported that bilateral pre-operant DBS reduced methamphetamine self-administration on treatment days (*p* < 0.05) and produced a significant within-group reduction in relapse-session methamphetamine seeking during Pavlovian spontaneous recovery (57% decrease, *p*< 0.01) [2].

#### Alcohol models

Alcohol models highlight the risk of paradoxical effects. While some studies suggest reduced ethanol preference after NAc DBS [24] and reduced intake after NAc shell inactivation/DBS in alcohol-preferring rats (e.g., 200 µA DBS reduced ethanol consumption by 35–59% on multiple days, *p*< 0.0018) [61], Hadar et al. reported that NAc DBS augmented relapse-like drinking in alcohol-dependent rats and increased alcohol-induced dopamine release, suggesting that stimulation may enhance alcohol reward under dependence conditions [21]. These divergent findings support state-dependent DBS effects that may differ between dependence phenotypes.

#### Parameter optimization and mechanistic studies

Mechanistic/parameter-optimization work suggests DBS is not a unitary intervention and may itself be rewarding. Evans et al. demonstrated that NAc stimulation patterns influence preference and reward-seeking, with animals preferring paradigms delivering higher pulse counts, and showing stimulation-related reward signals. [8] Such results have ethical relevance, raising theoretical concerns about reinforcement or misuse potential if stimulation produces euphoria-like effects [8]. These findings also argue for a more granular approach to target selection. “NAc DBS” or “VC/VS DBS” should not be treated as anatomically uniform interventions: core versus shell, dorsal versus ventral contacts, and relative engagement of ALIC fibers may plausibly produce different effects on craving, extinction, impulsivity, mood, and adverse activation. Similarly, targets outside the NAc/VC/VS region, including STN, mPFC/infralimbic cortex, anterior cingulate cortex, medial forebrain bundle, and extended amygdala/bed nucleus of the stria terminalis pathways, may map onto different relapse mechanisms such as reward salience, habit formation, negative reinforcement, stress reactivity, or impaired cognitive control. Emerging connectomic DBS work in other neuropsychiatric and movement-disorder fields supports the concept of therapeutic “sweet spots” and adverse-effect “sour spots,” in which small differences in stimulation location or fiber engagement can correlate with beneficial versus detrimental outcomes. Future SUD DBS trials should therefore report electrode localization, volume of tissue activated/e-field models, directional contact use when available, and connectivity to prefrontal, cingulate, amygdalar, hippocampal, and thalamic nodes. This would allow efficacy and adverse events to be mapped onto circuit engagement rather than treated as target-wide effects [25].

Additional preclinical work further demonstrates that neuromodulation effects in addiction models are highly dependent on both the stimulation target and the behavioral context. Across morphine CPP paradigms, dorsal–ventral distinctions within the ventral striatum shape the magnitude of DBS-induced extinction effects, and frequency-dependent responses highlight the importance of stimulation parameters [38]. Complementary studies show that stage-specific NAc stimulation can either facilitate or disrupt extinction and reinstatement processes, showing the temporal sensitivity of these circuits [64]. Mechanistic work further reveals that mPFC-NAc pathway modulation engages molecular signaling programs such as Fibroblast Growth Factor 1/Fibroblast Growth Factor Receptor 1 that contribute to durable behavioral change [16]. Electrophysiological evidence from mPFC-driven modulation of NAc ensembles provides additional support for circuit-defined, target specific effects in morphine CPP [6]. Beyond opioid paradigms, emerging strategies such as STN stimulation in alcohol-related behaviors broaden the translational relevance of these approaches and illustrate how distinct nodes within the addiction network can be leveraged to alter maladaptive reward-seeking [55].

### Risk of bias assessment and data synthesis

We did not perform a formal, tool-based risk of bias assessment because the clinical evidence base is dominated by case reports, small case series, and uncontrolled feasibility cohorts, for which standard instruments (e.g., Cochrane RoB2 for randomized trials, ROBINS-I/Newcastle–Ottawa for observational comparative studies, etc.,) are either not applicable or would generate largely uniform “high/unclear risk” ratings driven by study design rather than study conduct. In this context, assigning numerical or categorical risk-of-bias scores can give a false impression of precision and does little to discriminate between studies that differ mainly in reporting detail, follow-up intensity, and co-interventions rather than in core design features such as randomization and blinding. Instead, we used a structured qualitative appraisal aligned with the aims of this review (signal synthesis and trial-design implications rather than pooled effect estimation). Specifically, we extracted and considered study features most likely to introduce bias in neurosurgical SUD research such as patient selection and setting, concurrent treatments and monitoring intensity, outcome definition and verification, completeness and duration of follow-up, presence of blinded phases or delayed-activation designs, stimulation/programming transparency, and adverse-event ascertainment. We weighted interpretation accordingly, treating uncontrolled reports as hypothesis-generating while giving greatest inferential weight to controlled human evidence. For preclinical studies, formal scoring was similarly limited by heterogeneity in paradigms and frequent under-reporting of randomization/blinding/attrition. Animal findings were therefore used to inform mechanistic plausibility and parameter/state dependence rather than support quantitative conclusions about efficacy. This structured qualitative approach was used to avoid overstating certainty while still making differences in interpretive weight transparent across randomized, open-label, case-based, lesion, and preclinical evidence.

## Discussion

The neurosurgical literature in substance use disorders is still small, heterogeneous, and uneven in methodological rigor, but provides a coherent framework. Across substances, the most consistent signals are not instant abstinence switches but shifts in intermediate processes that sit upstream of relapse: craving, cue reactivity, affective tone (including anhedonia), and, more broadly, how reward and salience are assigned. This matters because the field has often leaned on binary end points (e.g., time to first use) that are easy to operationalize but may not map cleanly onto the way circuit interventions change behavior in chronic relapsing conditions. In parallel, DBS and lesion data reinforce that ventral striatal interventions are unlikely to be behaviorally “specific” in the narrow sense; they may alter motivation and reward processing across drug and non-drug domains. The practical implication is that next-generation trials should be designed to measure mechanism-relevant dimensions and meaningful functioning, rather than focusing only on lapses and toxicology.

### Alcohol use disorder

Alcohol use disorder offers the clearest reminder of why endpoints and mechanistic expectations matter. The randomized, double-blind trial of NAc stimulation remains the most rigorous dataset, and it importantly did not show separation on the primary time-to-first-drink endpoint during the blinded phase [1]. That finding should temper overly confident conclusions drawn from early open-label reports, but it also should not be misread as a simple refutation of clinical value. The same study suggests that stimulation can still matter in ways that are clinically recognizable, by shaping patterns of drinking and symptom dimensions that drive relapse risk (craving, anhedonia), even if an initial lapse is not prevented [1].

The distinction is not semantic. “First drink” is a brittle metric in a disorder where brief lapses are common, and where outcomes that patients and clinicians value, such as fewer heavy drinking episodes, more abstinent days, reduced compulsive drive, and improved affective tone, may be the more realistic first targets of circuit-based intervention. The open-label pilot, early case reports, and longer follow-up series fit comfortably with that framing: benefits, when present, tend to look like sustained reductions in consumption and compulsivity in subsets of individuals rather than uniform, immediate abstinence across cohorts [5, 31, 41, 42, 56]. The larger takeaway is that alcohol DBS studies may be better served by primary outcomes that reflect relapse vulnerability and functional recovery, with abstinence analyzed as an important but not exclusive marker of success.

### Opioid use disorder and heroin dependence

The opioid literature strengthens the interpretation that DBS may operate most reliably on proximal relapse drivers, particularly craving and cue reactivity, while long-term abstinence remains more variable and context-dependent. Across cohorts and case-based reports, craving reduction is a recurrent theme, and when abstinence is achieved it often co-occurs with broader stabilization in sleep, mood, or stress physiology, which are domains that are not incidental in OUD relapse risk [4, 36, 45]. This is one reason it may be misleading to treat abstinence alone as the “true” outcome and everything else as secondary; in refractory OUD, a credible circuit intervention may first show itself by lowering the intensity and intrusiveness of craving, reducing cue-induced destabilization, and enabling engagement in recovery behavior.

A second theme in OUD is that “NAc DBS” is not one intervention. Programming, contact selection, and individual differences in acute responses appear to matter, and several reports are most valuable precisely because they illustrate that outcome can hinge on how stimulation is delivered and which portions of the VS/ALIC region are engaged [4, 52]. That observation pushes the field away from viewing DBS as a fixed dose and toward thinking of it as a titratable, personalized therapy whose effects should be verified against mechanistic readouts when possible.

In that regard, within-subject mechanistic approaches are especially instructive. They shift the evidentiary center of gravity from distal endpoints that are multiply determined (abstinence vs relapse) to measurable processes that can plausibly link stimulation to behavior (e.g., cue-reactivity signatures) [44]. Even if the clinical literature is not yet large enough to adjudicate optimal targets and parameters, these designs at least offer a path toward explainable outcomes, such as why a given setting worked for a given patient, rather than relying on post hoc narratives.

At the same time, the OUD DBS evidence base still faces the standard interpretive challenge of early neurosurgical psychiatry trials: uncontrolled protocols often include intensive monitoring, frequent clinician contact, and structured support, each of which can independently improve outcomes [36, 45]. This does not invalidate the clinical signals, but it does mean that controlled designs, standardized co-interventions, and blinded phases (where feasible) remain essential if the field is to separate stimulation-specific effects from the benefits of enhanced care.

### Stimulant use disorders

The stimulant literature remains the least mature clinically, but it contributes two crucial insights that should shape the next generation of trials. First, the literature highlights that the relevant circuitry may not be identical across drugs, and that it may be premature to assume that a single VS target will generalize [14, 58]. Second, it brings the adverse-effect profile into sharp focus: stimulation-induced activation (including hypomanic symptoms under some settings) is not just a side note in stimulant populations, it can be a limiting factor and a confound, given baseline vulnerability to impulsivity, sleep disruption, and affective instability [58].

The limited evidence exploring more ventral/extended limbic targets (e.g., accumbens shell/bed nucleus of the stria terminalis) is notable because it suggests alternative mechanistic hypotheses, perhaps more directly engaging stress and negative-affect circuitry that can maintain stimulant use even when hedonic reward wanes [14]. Meanwhile, methamphetamine case reports raise provocative biological observations, such as changes in striatal dopamine transporter levels after prolonged stimulation, but remain too sparse for firm inference [13, 67]. The overarching point is that the stimulant literature argues for substance informed neurobiology: trials should be explicit about which relapse pathway is being targeted (reward salience, habitual responding, negative reinforcement, affect dysregulation), rather than treating “addiction” as a single circuit problem.

### Mechanistic and ethical lessons from the ablation literature

The ablation literature serves as both precedent and caution. Mechanistically, lesion approaches are hypothesized to reduce relapse vulnerability by interrupting ventral striatal and ventral capsular pathways that assign motivational salience to drug cues, mediate reinforcement learning, and link limbic drive with frontostriatal action selection. In NAc-centered procedures, the intended effect is not simply “removal” of a craving center, but disruption of distributed mesocorticolimbic loops involving the NAc, orbitofrontal and medial prefrontal cortex, anterior cingulate cortex, amygdala, hippocampus, and thalamic projections. This rationale overlaps with DBS in that both modalities target reward-control circuitry; however, their biological and clinical effects may diverge. DBS may act through reversible modulation of firing patterns, oscillatory activity, synaptic plasticity, and network communication, whereas ablation produces irreversible tissue or fiber disruption and cannot be titrated after the lesion is made [11, 32, 63].

This distinction is particularly important in SUDs because ventral striatal circuits contribute to drug reward, natural reward, motivation, affect, and decision-making. Lesioning may reduce drug-related salience in some patients, but it may also alter non-drug reward processing, hedonic tone, impulsivity, sexual function, appetite, or broader motivation [59]. Therefore, ablation data should be framed as a cautionary historical comparator and as evidence that the target network is behaviorally powerful, not as evidence that irreversible procedures and DBS carry the same risk–benefit profile.

Where the ablation literature becomes particularly informative for contemporary DBS is in its documentation of non-drug behavioral and motivational changes after VS intervention [59]. That line of evidence is a reminder that manipulating these networks may shift valuation and motivation globally, not just drug salience. For DBS trials, the implication is concrete: studies should prospectively assess hedonic tone, apathy, impulsivity, and quality of life, and should track non-drug reward domains and other addictive behaviors. Without these measures, clinically meaningful tradeoffs could remain invisible, particularly in short trials focused narrowly on substance-specific endpoints.

### What preclinical studies add to interpretation and trial design

The preclinical literature does not “prove” efficacy, but it materially strengthens the field’s ability to design interpretable human trials by clarifying parameter dependence and candidate mechanisms. Across substances, rodent work repeatedly shows that DBS effects depend on (1) target subregion, (2) stimulation frequency and pattern, and (3) timing relative to extinction/reinstatement or relapse-like states. For example, multiple cocaine models suggest that NAc stimulation can reduce reinstatement under some conditions, yet effects vary by whether stimulation is continuous or patterned [18, 19, 22, 26, 47, 53, 54]. Ethanol studies illustrate the same principle from the opposite direction: in certain relapse-like paradigms, NAc DBS can augment alcohol intake, highlighting that neuromodulation can plausibly be pro-relapse depending on state and context [21, 24]. This is not a minor nuance; it means that human trials must be explicit about whether stimulation is intended to support abstinence maintenance, dampen cue-reactivity during early recovery, facilitate extinction learning, reduce negative affect, or accomplish multiple goals via adaptive programming. This point deserves special emphasis for trial design. In alcohol use disorder, the strongest clinical signal comes from NAc DBS, yet preclinical alcohol-dependent models also show that stimulation can worsen relapse-like drinking under some conditions [1, 21]. Rather than weakening the rationale for DBS, this underscores the need to define the intended therapeutic state: early abstinence, cue exposure, withdrawal/negative affect, extinction learning, or relapse prevention. It also supports careful parameter titration, prospective monitoring for increased craving or use, and consideration of adaptive or state-contingent stimulation paradigms rather than assuming continuous high-frequency stimulation will be uniformly therapeutic.

Another key preclinical insight is that “reward circuitry” is not confined to the NAc. Several morphine studies targeting cortical and limbic regions (e.g., mPFC, anterior cingulate) report modulation of CPP, reinstatement, and cognitive/affective measures, suggesting that targeting cognitive control and affect regulation circuits may be an alternative or complementary route to reducing relapse vulnerability [6, 10, 16, 34, 35, 38, 64]. Importantly, these studies encourage a “network” view consistent with emerging human findings: neuromodulation may act by shifting large-scale fronto-striatal balance rather than by locally silencing a single nucleus.

Finally, preclinical work on stimulation patterning adds a practical translational level. Studies examining intermittent versus continuous paradigms, and demonstrating that stimulation itself can carry reinforcing properties depending on parameters, highlight why human trials should not treat parameter selection as an afterthought [2, 8, 55, 61]. In short, the animal literature strengthens the argument that future clinical trials should incorporate mechanistic endpoints and adaptive approaches rather than assuming one-size-fits-all continuous high-frequency stimulation.

### Safety signals and ethical implications

The results emphasize that surgical and device-related complications, while not ubiquitous, are clinically meaningful and must be incorporated into consent and trial governance. Across reports, infections requiring intervention and stimulation-related neuropsychiatric changes are the recurring high-salience risks. For example, device infection necessitating explantation occurred in the alcohol DBS pilot experience [5], and compliance challenges can lead to explantation in the context of OUD feasibility work [45]. Stimulation-related activation, ranging from transient mood elevation and insomnia during programming to more concerning hypomanic symptoms, appears across multiple opioid and stimulant reports, with the crack cocaine STN case demonstrating how hypomania can derail planned blinded assessments [4, 58]. In the context of SUDs, these risks carry added ethical weight because they intersect with baseline impulsivity, psychiatric comorbidity, and the real-world harms of relapse.

The clinical relevance of these adverse events should be judged against the severity and refractoriness of the treated illness. In patients with severe, treatment-refractory SUDs and repeated overdose, medical complications, or failed evidence-based therapies, reductions in craving, heavy-use days, or cue reactivity may be meaningful even without immediate complete abstinence. However, such benefits justify invasive intervention only if they are durable, functionally meaningful, and accompanied by careful monitoring for surgical complications, device-related burden, neuropsychiatric activation, suicidality, impulsivity, apathy/anhedonia, cognitive change, and shifts in non-drug reward behavior. This benefit-risk threshold is especially high for lesion approaches because adverse motivational or neuropsychological effects cannot be reversed by changing stimulation settings or turning off a device. These considerations also affect how newer lesioning technologies should be framed. MR-guided focused ultrasound (MRgFUS) may reduce procedural burden by enabling incisionless, MRI-guided lesioning without craniotomy or implanted hardware, but it remains an ablative and irreversible intervention once a lesion is made. Therefore, MRgFUS changes the invasiveness profile, but it does not eliminate the ethical issues associated with permanent manipulation of reward circuitry. A recent post-search case report of bilateral NAc MRgFUS for severe AUD described reduced craving, 12-month abstinence, and no serious adverse effects, but this single case should be interpreted as feasibility-generating rather than efficacy-establishing evidence [60].

Ethically, SUD neurosurgery is distinguished not only by the usual challenges of psychiatric DBS (identity, authenticity, affective change), but also by the risk of coercion and structural vulnerability. Many individuals with severe SUD have legal, financial, and interpersonal pressures that could distort voluntariness. The ablation experience in particular emphasizes why reversibility and long-term monitoring matter: irreversible lesions in reward circuitry can plausibly affect natural motivations and thus the patient’s broader life, not simply drug use [59]. Accordingly, the strongest ethical posture for future trials includes (1) independent consent monitors, (2) explicit protections against legal or institutional coercion, (3) robust post-operative psychosocial care commitments, and (4) fairness considerations to prevent neurosurgery from becoming either an inaccessible boutique intervention or a disproportionately applied intervention in marginalized groups.

### Roadmap for next-generation trials

The results suggest several practical, method-focused changes that would make these studies easier to interpret and more useful clinically. One place to start is with endpoints that fit with what DBS is actually supposed to do. If the goal is to dampen cue reactivity and compulsive drive, not guarantee perfect abstinence, then it makes sense for primary outcomes to include things like proportion of abstinent days, number of heavy-use days, validated craving/compulsitivity measures, and cue-reactivity indices, instead of treating “time to first lapse” as the only meaningful marker. The alcohol RCT makes this point clearly, a trial can miss its primary endpoint and still show secondary effects that are better aligned with the mechanism and arguably more clinically informative [1]. Another key step is to build mechanistic assessments into the design from the outset. Approaches that use electrophysiology or imaging to guide personalization, such as combining cue-reactivity tasks with intracranial signatures, should be specified prospectively rather than introduced later as explanatory add-ons [44, 52]. Along the same lines, metabolic imaging patterns linked to sustained abstinence in early OUD work point to candidate biomarkers that can be carried forward and tested directly in future trials [45]. In addition, co-interventions need to be handled more rigorously; psychosocial treatment “dose,” medication-assisted treatment, toxicology testing schedules, and relapse definitions should be prespecified and reported in a standardized way, because abstinence rates are not comparable across cohorts when these elements vary or go unreported. Finally, the field needs more sham-controlled comparison and longer follow-up when feasible. Blinding is difficult in DBS and can be undermined by stimulation side effects of obvious clinical changes, but without carefully monitored sham conditions and follow-up periods that match the chronic, relapsing nature of SUDs, it will be hard to draw firm conclusions about efficacy and durability. Next-generation trials should also incorporate newer neuromodulation technologies. Adaptive DBS is especially relevant for SUDs because craving, cue reactivity, withdrawal, stress, and relapse risk are state-dependent rather than constant. Adaptive DBS work in Parkinson disease demonstrates the feasibility of closed-loop stimulation that responds to changing neural states, and biomarker-informed OUD DBS demonstrates the feasibility of linking stimulation parameters to individualized craving circuitry [43, 44]. A current individualized adaptive DBS trial in OUD is evaluating whether invasive brain mapping can guide personalized stimulation to reduce craving and opioid use [40]. These approaches may help reduce unnecessary stimulation, avoid stimulation-induced activation, and test whether closed-loop or state-contingent stimulation is superior to continuous DBS.

Focused ultrasound technologies should be discussed separately by mechanism. MRgFUS is best conceptualized as a less invasive method for creating an irreversible lesion, whereas low-intensity focused ultrasound (LIFU) is a non-ablative neuromodulation strategy that may transiently modulate deep reward circuitry without implanted hardware. Although LIFU was outside the eligibility criteria of this invasive neurosurgical review, early SUD studies suggest feasibility and tolerability. A 2023 pilot study of bilateral NAc LIFU in four participants reported no structural MRI changes and acute craving reductions, and a 2025 open-label study in eight participants with severe OUD reported no serious device-related adverse events, sustained reductions in cue-induced opioid craving, and urine-toxicology-supported decreases in substance use over 90 days [37, 46]. These early data support LIFU as a future comparator or stepwise intervention before invasive DBS or irreversible lesioning, but larger sham-controlled trials are needed.

### Limitations

This systematic review has several limitations. The primary limitation of this manuscript is the immaturity and heterogeneity of the underlying human evidence base. Across substances, most clinical reports are case studies or small, open-label cohorts with substantial variation in targets (and target subregions), stimulation parameters, programming intensity, follow-up duration, and concurrent treatments (psychosocial interventions and pharmacotherapy), making results difficult to compare directly and highly vulnerable to confounding. Invasive SUD trials are also particularly susceptible to non-specific effects such as enhanced monitoring, frequent clinician contact, expectancy, perioperative stabilizations, and regression to the mean, while DBS-specific challenges (e.g., stimulation-related mood/activation effects) can undermine blinding and complicate causal attribution. Outcome reporting is inconsistent, with variable relapse definitions and uneven use of objective toxicology schedules; safety reporting is similarly nonuniform and may under-capture neuropsychiatric tradeoffs. Finally, because ventral striatal/limbic interventions can plausibly affect motivation broadly, the relative undermeasurement of domains such as hedonic tone/anhedonia, apathy, impulsivity, cognition, and quality of life limits the ability to characterize benefit-risk profiles beyond substance-focused endpoints. Another major limitation is that this review’s synthesis is necessarily narrative and hypothesis-generating, rather than quantitatively definitive. Given the heterogeneity in study designs, endpoints, and reporting quality, meta-analytic pooling is not currently well-justified, and formal risk-of-bias assessment is constrained by incomplete methodological detail in many reports. Publication bias is also a concern as the literature is dominated by positive case reports and single-center experiences, and the generalizability of findings is limited by highly selected patient populations able to engage in intensive longitudinal follow-up. In addition, ablation studies and DBS trials arise from different clinical, regulatory, and ethical contexts, while preclinical models only partially capture the social, psychiatric, and environmental determinants that shape human relapse. Collectively, these limitations suggest that current conclusions support clinical plausibility and can help guide trial design, but they do not yet establish definitive efficacy, optimal targets, or programming strategies across SUD subtypes. Finally, rapidly emerging technologies such as adaptive DBS, MRgFUS lesioning, and LIFU neuromodulation are evolving faster than the current clinical evidence base. Because the formal search ended on September 9, 2025 and excluded non-invasive neuromodulation, post-search reports and LIFU studies were discussed only as future directions and were not included in the PRISMA count or primary clinical efficacy synthesis. One preprint captured within the original search was updated to its peer-reviewed publication during revision.

## Conclusion

Neurosurgical modulation of reward circuitry, most commonly targeting the NAc and adjacent VC/ALIC, has produced encouraging but still preliminary signals in severe, treatment-refractory substance use disorders. Across alcohol, opioid/heroin, and stimulant use disorders, the clinical literature describes reductions in craving and, in some cases, sustained abstinence or marked decreases in consumption. However, the current evidence remains dominated by small uncontrolled studies and single-case reports, and even the strongest controlled data suggest that benefits may be more apparent on secondary measures of drinking behavior, craving, anhedonia, and cure reactivity than on time-to-relapse-style primary endpoints. Taken together, the human evidence supports clinical plausibility and trial design, but not yet definitive efficacy or generalizable target selection across SUD subtypes. DBS and lesion approaches should therefore be advanced along different evidentiary and ethical pathways. DBS warrants further study in sham-controlled, biomarker-informed, and potentially adaptive designs because it is programmable and can be modified in response to benefit or adverse effects. Lesion approaches, including newer incisionless methods such as MRgFUS, may reduce procedural burden but remain irreversible and should require especially strong evidence of durable benefit, comprehensive neuropsychological monitoring, and rigorous consent safeguards before broader clinical adoption.

## Supplementary Information

Below is the link to the electronic supplementary material.

ESM 1 Supplementary Material 1 (DOCX 7.67 KB)

## Data Availability

No new primary data, patient-level data, or biospecimens were generated or collected for this systematic review. All data analyzed in this manuscript were extracted from previously published studies identified through the systematic search of PubMed, PubMed Central, Embase, and Scopus. The extracted study-level data supporting the findings are included in the manuscript and its supplementary materials; additional extracted data are available from the corresponding author upon reasonable request.
