# Supplementary material for: Rewiring reward in addiction: a systematic review of human evidence for deep brain stimulation and lesion approaches in substance use disorders
Source: Acta Neurochir (Wien). 2026 Jul 14;168(1):232. doi: 10.1007/s00701-026-06979-0 (PMC13649863; doi:10.1007/s00701-026-06979-0)
Supplement: Supplementary file 1 [file 701_2026_6979_MOESM1_ESM.docx]

Supplemental Table 1: Search Terms

| **Database** | Full Search Syntax | Number of Search Results |
| --- | --- | --- |
| PubMed | "deep brain stimulation" OR "DBS" OR "stereotactic surgery" OR "capsulotomy" OR "lesion" OR "ablation") AND ("nucleus accumbens" OR "ventral striatum" OR "ventral capsule" OR "subthalamic nucleus" OR "medial forebrain bundle") AND ("substance use disorder" OR "addiction" OR "drug dependence" OR "alcohol use disorder" OR "opioid use disorder" OR "cocaine" OR "methamphetamine" OR "stimulant use disorder" OR "nicotine dependence" OR "tobacco use disorder"). | 19 |
| Embase | ('deep brain stimulation'/de OR 'dbs':ti,ab OR 'stereotactic surgery'/de OR neurosurgery:ti,ab) AND ('substance use disorder'/exp OR 'addiction'/de OR 'alcohol dependence'/de OR 'opioid dependence'/de OR 'cocaine dependence'/de OR 'nicotine dependence'/de) AND ('nucleus accumbens'/de OR 'ventral striatum':ti,ab OR 'ventral capsule':ti,ab OR 'subthalamic nucleus'/de OR 'medial forebrain bundle':ti,ab) AND [humans]/lim | 395 |
| Scopus | ( ( "deep brain stimulation" OR DBS OR "stereotactic surgery" OR neurosurgery ) AND ( "substance use disorder" OR addiction OR "alcohol dependence" OR "opioid dependence" OR "cocaine dependence" OR "nicotine dependence" ) AND ( "nucleus accumbens" OR "ventral striatum" OR "ventral capsule" OR "subthalamic nucleus" OR "medial forebrain bundle" ) ) | 254 |
| PubMed Central | ("deep brain stimulation"[MeSH] OR "stereotactic neurosurgical procedures"[MeSH]) AND ("substance-related disorders"[MeSH]) AND ("nucleus accumbens"[MeSH] OR "subthalamic nucleus"[MeSH]) | 19 |
